# Women’s empowerment and intra-household gender dynamics and practices around sheep and goat production in South East Kenya

**DOI:** 10.1371/journal.pone.0269243

**Published:** 2022-08-04

**Authors:** Kennedy O. Ogolla, Judith K. Chemuliti, Mariah Ngutu, Winnie W. Kimani, Douglas N. Anyona, Isaac K. Nyamongo, Salome A. Bukachi

**Affiliations:** 1 Biotechnology Research Institute, The Kenya Agricultural Livestock Research Organization, Kikuyu, Kenya; 2 Department of Anthropology, Gender and African Studies, University of Nairobi, Nairobi, Kenya; 3 The Co-operative University of Kenya, Nairobi, Kenya; International Food Policy Research Institute, UNITED STATES

## Abstract

Small ruminant production facets like decision-making, ownership, labour allocation, access to- and control over assets are gendered. This study investigates intra-household gender dynamics and practices around sheep and goat production among smallholder farmers in South East region of Kenya. A quantitative study was conducted on 358 dual-headed (married) households to generate gender-disaggregated data on ownership, decision-making and labour allocation around small ruminant production. Qualitative data was collected through focused group discussions to bring out the community perspectives. From the findings, the average number of small ruminants owned by the households as reported by men was slightly higher than women. The average number of small ruminants solely owned by men was significantly higher than by women. Men reported a relatively higher number of jointly owned small ruminants compared to women. More women than men reported that they could give as a gift, sell-off and slaughter jointly owned small ruminants without consulting their spouses. Small ruminants were considered the most important livestock asset in supporting a household’s livelihood by relatively more women than men. Men had more decision-making autonomy over jointly owned small ruminants compared to women. Production tasks around small ruminants such as feeding, watering, selling milk and cleaning housing structures were mostly performed by the women. Qualitative data identified men as the de facto owners of small ruminants with a higher power position in making the important production decisions. The study offers three implications on the design of livestock interventions to empower women, the interventions should ensure that; 1) women are not just owners of livestock assets but also share power and decision-making rights in all aspects of production, 2) production labour is shared equitably between men and women and, 3) women access benefits from livestock production even when animals are owned by men.

## Introduction

Rearing of small ruminants (sheep and goats) supports livelihoods and wellbeing of many rural households in developing countries due to their productive and reproductive advantages [1, 2]. They are important sources of good quality meat and milk for household nutrition [3], require less land space and are more prolific compared to large ruminants. They are also easy to manage, perform well in most agro-ecological zones [3, 4], and their production is therefore a good entry point for development projects that seek to empower women and create sustainable community resilience through livestock production [5–8]. The concept of ‘power’ in ‘empowerment’ has not only changed over time but has also continued to be understood multifariously in different socio-cultural settings [9]. At a personal cognitive level, individuals understand ‘empowerment’ as the personal strides, strives and efforts that they have to make towards self-determination and self-efficacy [10–12]. However, the term ‘empowerment’ also has a multidimensional and relational aspects, being a factor of an individual’s interactions with other people, groups or institutions within or between societies [13–17]. Whereas an individual’s attainment of ‘empowerment’ is highly dependent on the changes taking place within the individual, the outcome is strongly influenced by how power relations play out in the individual’s interaction with others and society [9], as no one exists in a vacuum. Over time, individualistic and collectivistic (relational) paths towards ‘empowerment’ have given rise to different variations of ‘power’ that attempts to explain what is meant by being ‘empowered’ albeit at different levels. The five variations of power have been discussed comprehensively in literature as power through [9], power with [18], power over [19], power to [20], and power within [21].

Although these power variations are different, they share a common broad goal of expanding the capacity of an individual or groups of individuals to make free choices and to transform these choices into desired actions and outcomes as defined under the United Nations’ sustainable development goal no.5. Empowerment is therefore a process that encompasses the changes that move one from being ‘disempowered’ to ‘empowered’. Empowerment of rural women is critical in enabling them to reach self-determination and in ascribing them more autonomy to make choices, decisions, and have financial control [12]. Empowered women tend to be more productive and proactive, and are also able to contribute to the affairs of their community [17, 22]. Women contribute significantly to the productivity, income, nutrition, and health of their household members and their community at large [13, 15, 16]. However, while women complement the financial contributions of men in the households, it is often difficult to quantify their exact input; a big proportion of which goes unnoticed [23].

Globally, women make decisions to varying levels [13, 22, 24]. In SSA, women spend most of their time performing household chores owing to biased socio-cultural norms that impose such chores on them [25, 26]. Under traditional settings, women are not only expected to perform reproductive functions of childbearing but also caregiving [16, 26]. In many regions of sub-Saharan Africa (SSA), women are economically exploited and are either underpaid or unpaid for their labour [22]; also, the women are socially oppressed, ignored on legal issues, and lag behind in technological advances [22, 27]. Nonetheless, women remain the backbone of rural economies where they offer substantial agricultural labour but they seldom reap the financial benefits [28–30]. Evidence shows that the agricultural sector is underperforming in developing countries because of historical and structural socio-cultural norms and practices that hinder women from participating fully in agriculture [31]. These challenges accentuate the need for women empowerment.

The prevailing gender dynamics and practices around livestock production substantially influence the ability of intervention programs aimed at empowering women using livestock assets [32]. Gender dynamics and practices around decision-making, labour allocation, asset ownership, access to- and control over resources affect the success of women empowerment programs [23, 28]. Gender practices in livestock rearing are known to vary across regions, among different communities and depending on livestock species in question [33–35]. For instance, production of small ruminants in some parts of Kenya and its neighbouring countries is largely under the management of women with men preferring to keep the large ruminants [35–38]. Studies conducted in parts of Kenya have established that a large proportion of small ruminant production labour is offered by the women [33, 38]. The likelihood of the women owning small ruminants was also found to be higher compared to large ruminants [33, 38].

Women’s access to and control over productive resources, and their ability to access and use income from the sale of small ruminants are also influenced to a large extent by the existing socio-cultural norms [31, 39, 40]. Whereas the norms may favour women in some communities, they can be quite unbearable in others and this limits their ability to create wealth and make decisions on matters that affect their lives [22, 25]. For example, in a previous study, while Kenya’s “Ameru” women had full control over the sale of goats and goat products and in the use of income thereof [38], their counterparts from the "Kalenjin" community did not enjoy such liberties as they only had control over the evening milk which was mostly consumed at home while the men controlled sale of morning milk that went to formal markets [41]. Similar results have been reported in other countries [35, 42]. However, even where women appear to have control, empirical evidence suggests that men tend to take over control of small ruminant production when they become profitable [35, 42–44].

These gender dynamics and practices affect the success of intervention programs seeking to empower women through livestock production and it is therefore important to identify gender gaps and disparities that should be targeted or considered by interventions to uplift and empower women farmers. The paper uses empirical data from a dual-household survey to establish intra-household gender dynamics and practices around small ruminant production in Makueni county. The paper further documents spousal differences in reporting around small ruminant production activities and tasks. The key facets addressed relate to ownership, input into decisions, access to and control over income, labour allocation/participation in small ruminant production activities, participation in disease prevention and treatment, and selection of species and breeds to rear.

## Materials and methods

### Ethical approval

The study was approved by the Strathmore University Institutional Ethics Review Committee (SU-IERC0523/13). In addition, the project was licensed to conduct research in Makueni County by the National Commission for Science, Technology and Innovation (NACOSTI/P/19/1207).

### Study background and context

This study is part of an ongoing Gender Inclusive Vaccine Ecosystem (GIVE) (https://idl-bnc-idrc.dspacedirect.org/handle/10625/59232?show=full) action research project being implemented by the University of Nairobi (UON), Kenya Agricultural and Livestock Research Organization (KALRO) and the Cooperative University of Kenya (CUK). The project seeks to enhance the distribution and delivery systems for Newcastle disease and contagious caprine pleuropneumonia vaccines among smallholder farmers, as well as to increase women’s participation in the livestock vaccine value chain in the South-East region of Kenya. The study used the Women Empowerment in Livestock Index (WELI) measurement tool [45] to investigate intra-household gender dynamics and practices around small ruminant production.

### Women Empowerment in Livestock Index (WELI)

To overcome challenges previously encountered with quantifying women empowerment, and to demonstrate gender dynamics, trends and practices around livestock production when assessing impacts of intervention projects [46], and to allow within and between countries/projects comparisons, Women Empowerment in Agriculture Index (WEAI) was developed from a joint exercise by USAID, IFPRI and the Oxford Poverty and Human Development Initiative as a measure to assess women empowerment in agriculture [17]. A joint team from the International Livestock Research Institute (ILRI) and Emory University then building on WEAI, developed WELI, a comprehensive and standardized tool used to measure women empowerment and to investigate gender dynamics, gender trends and practices around livestock production [45]. The domains of interest in WELI include; 1) decisions around agricultural production, 2) decisions about nutrition, 3) access to and control over productive resources, 4) control over and use of income, 5) access to and control of opportunities and, 6) workload and control over own time [45]. The WELI comprises two components; the quantitative part which is the main component and the qualitative part which complements the quantitative survey by providing in-depth information on contextual understanding of empowerment and gendered practices.

The two components of WELI have been used in several studies either jointly or individually since the conception of the tool [46–48]. The present study used a mixed quantitative and qualitative WELI to investigate intra-household gender dynamics and practices around small ruminant production in a setup of a developing country. While the authors acknowledge that the strength of WELI lies in comparisons of project impact across different populations (settings) and in tracking change over time within populations both of which require longitudinal data, the aim of the present paper was not to measure women empowerment per se but to investigate gender dynamics and practices around small ruminant production using point data from a baseline survey that was carried out at the onset of the GIVE project. Cross-sectional data is therefore used in this paper to investigate; 1) intra-household gender differences in control and decision making around small ruminant production, 2) intra-household gender differences in control and decision-making regarding jointly and solely owned small ruminants, 3) spousal differences in reporting ownership, control and decision-making around small ruminants, and 4) gender disparities between what people commonly believe (from our FGD data) versus individual perceptions at the household levels (quantitative data).

### Study area and selection of study sites

**Fig 1:**
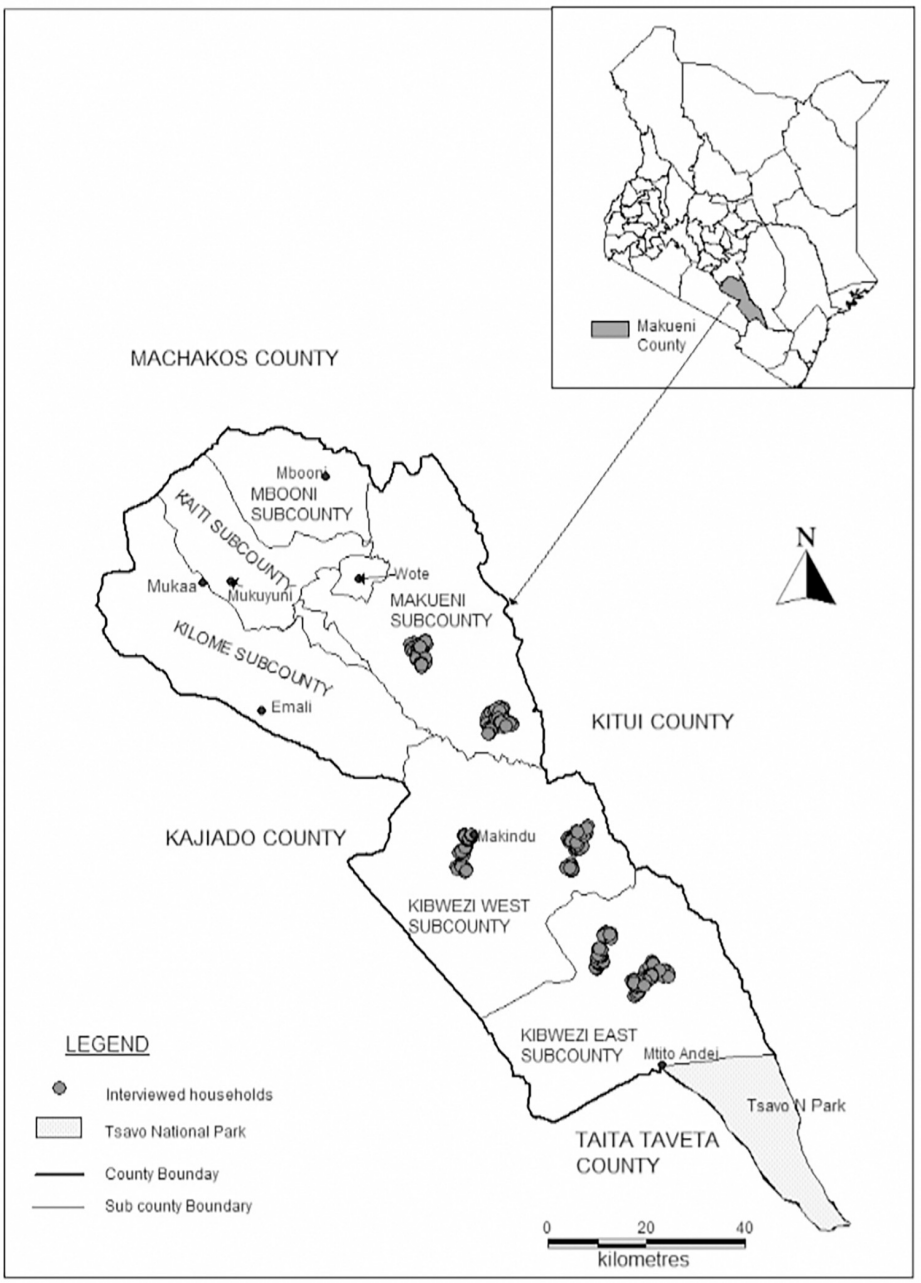
A map of the study area and households interviewed (made with Natural Earth).

The study was conducted in Makueni County located in South East Kenya. The county is largely arid and semi-arid land with marginal crop farming. It is however, ideal for small ruminant production. The study was carried out in three purposively selected administrative sub-counties namely: Makueni, Kibwezi East, and Kibwezi West, located in the lower more arid areas of the county. Questionnaires were administered in two randomly selected administrative wards in each sub-county: Kibwezi East (Masongaleni and Mtito Andei wards), Kibwezi West (Makindu, and Kikumbulyu North wards) and Makueni (Kitise and Kathonzweni wards). These sub-counties have established small ruminant production systems with most households keeping at least a few goats and/or sheep. The county covers an area of 8169.8 km^2^ with a population of 987,653. It has a total of 244,669 households each having an average of 4 members with a population density of 121 people per sq. km according to the 2019 national household and population census. The county lies between Latitude 1^o^ 35’ and 32^o^ 00’ south and Longitude 37^o^ 10’ and 38^o^ 30’ east. The annual rainfall ranges between 250 mm—400 mm p.a in the lower regions of the county and 800 mm– 900 mm p.a in the higher regions. The map of the study site with sampling locations is presented in **Fig 1**.

### Study design, study population and selection of households

This was a cross-sectional study. The target population comprised sheep and/or goat farmers residing in the six wards within Makueni county. The study population comprised of 358 randomly selected dual-headed (married) households that kept small ruminants either at the time of the survey or 3 months before the survey. The ultimate sampling unit in the present study was the household. For this study, a household was defined as a set of related or unrelated individuals habitually sharing the same dwelling (whether it is their main residence or not) and who had a joint budget. The study was conducted between April and May 2020.

### Administration of WELI questionnaire

The WELI questionnaire was digitized in Open Data Kit (ODK Collect 1.28) software and administered using tablets by project-trained enumerators who also took part in its pre-test a *priori*. The questionnaire was administered to both the husband and wife in each household to gather their independent views and perceptions on small ruminant production practices and ownership. In the few households where the husband or wife was not available, the most senior adult man or woman was interviewed instead. Two enumerators were deliberately assigned a household to enable them interview the man and woman separately but concurrently.

### Gender aspects assessed and definition of terms

**Fig 2:**
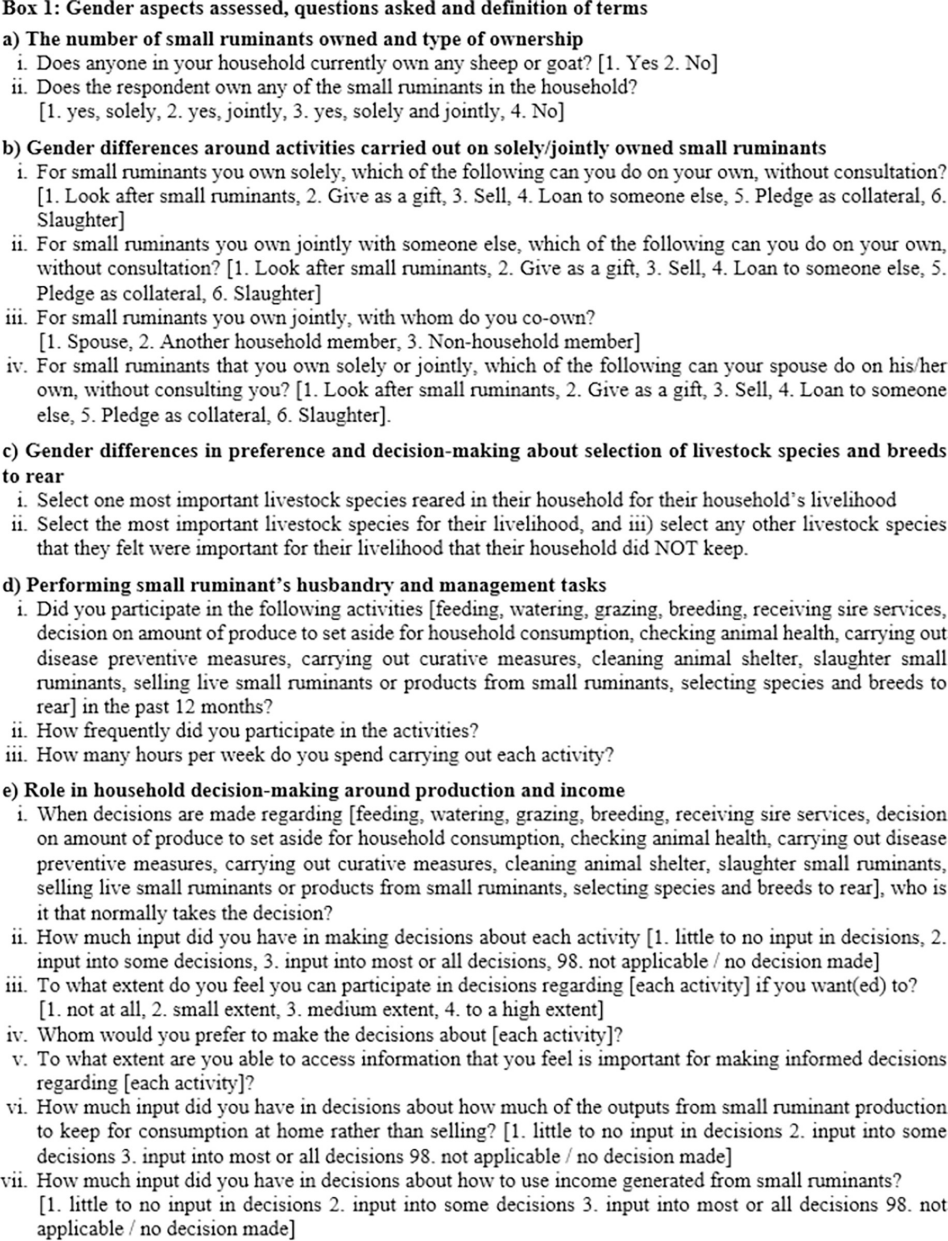
The gender aspects assessed and questions that the respondents were asked.

The respondents were assessed on the gender aspects of small ruminant production summarized in **Fig 2**.

In this study, giving small ruminants as gift entailed transferring the animal(s) to another individual for no direct return, whereas selling off entailed transferring the animal(s) for a direct return either in cash or kind. Giving small ruminants as loan entailed transferring the animal(s) to another individual on condition that the transferred animal(s) will be returned in the future or its equivalent value while pledging as collateral meant the animal(s) was given away as security when procuring a loan and is only returned when the loan is fully settled. The definitions of the task assessed are described in detail in the WELI instructional guide provided as S1 Appendix while the complete WELI quantitative questionnaire is provided as S2 Appendix. The questionnaire was administered in three languages: mainly Kamba and Swahili, and on rare occasions, English depending on the choice of the respondent and the responses captured in English in ODK Collect 1.28 software.

### Focus Group Discussions (FGDs)

Twenty-three FGDs sessions (13 women-only and 10 men-only) were conducted in randomly selected locations in 6 administrative wards to allow for a deeper understanding of the quantitative data and to establish overt and subtle differences in the group and individual perspectives on diverse gender aspects around small ruminant production. The sessions were distributed as follows: Makueni sub-county (9), Kibwezi West (8) and Kibwezi East (6). There were at least 2 women-only and 1 men-only FGDs per ward each comprising of 8 to 12 participants.

The participants were purposively selected based on their practice of small ruminant production. Household heads or spouses that had at least one (1) goat or sheep at the time of study or 3 months prior and were willing to participate in the discussions for the agreed duration were included. Village-level community mobilizers helped in the identification of potential FGDs participants. The discussions were conducted in the local Akamba dialect and moderated by trained research assistants well versed in the local language and socio-cultural context of the participants. Using an already pre-tested FGD guide, the participants were asked a set of questions and probing to obtain as detailed information as possible. Each session was facilitated by two moderators; one asked the questions, listened, and facilitated the discussions while the other took notes. Additionally, voice recorders were used to record all the discussions. The insights from FGDs were used to triangulate information obtained from the quantitative household survey.

### Informed consent

Informed consent was obtained from each respondent before participation in the study. The consenting process involved providing the respondent with adequate information about the study, outlining the possible benefits and consequences of participating in the study, responding to the respondents’ questions, making it clear that the respondent is free to discontinue the interview at any point in time, providing ample time for the respondent to make a decision, and finally obtaining the subject’s voluntary agreement, by way of a signature or thumbprint on the informed consent form.

### Data management and analysis

Data captured electronically in ODK Collect were exported into Microsoft Excel spreadsheet 2016 for cleaning and further coding. Coded data were then imported into STATA version 14 for analysis of descriptive statistics and tests of association. Descriptive statistics were presented as frequencies and means ± standard deviations. Tests of statistical significance were carried out using Pearson Chi-square two-sided (χ2) for two or more categorical data comparisons and independent t-tests (two-tailed) to determine the association between categorical and continuous datasets. Frequencies and proportions were used to show gender differences in participation and decision-making in various small ruminant production activities. The proportions were calculated based on the number of respondents that gave a response to the question (as the denominator) i.e number of respondents that reported they could carry out an activity divided by the total number of respondents that answered the question (sum of those that stated they could do the activity and those that reported they could not). For qualitative data, the audio files and notes were transcribed and translated into English transcripts for analysis. The transcripts were reviewed against the audio files for quality. One-third of clean transcripts were read through by 3 researchers to identify emerging themes and codes which were used to inform the framework used for coding on NVIVO qualitative analysis software version 12. The summaries from the coded qualitative data were categorized based on the main themes informed by the study objectives. Key phrases were quoted verbatim to reflect participants’ views, beliefs, and perceptions.

## Results

### Demographic characteristics of quantitative survey respondents

A total of 716 respondents (358 men and 358 women) from 358 households met the inclusion criteria and were interviewed. Of the interviewed households, 350 (97.8%) were dual-headed households with a husband and a wife while 8 (2.2%) only had a woman head and therefore, senior-most men in the households were interviewed instead. The majority of the respondents (676, 94.4%) were interviewed alone while the rest were either interviewed with adult women present (13, 1.8%), adult men present (2, 0.3%), adults of both gender present (1, 0.1%), children present (23, 3.2%), or adults of both gender and children present (1, 0.1%). Disaggregation of the survey respondents by administrative sub-counties, wards and gender is presented in **Table 1**.

**Table 1:** Survey respondents disaggregated by administrative sub-counties, wards and gender.

No.	Sub-county	Ward	Sex		Total	
			Men	Women	Freq.	Percent. (%)
1.	Makueni (238, 33.2%)	Kathonzweni	60	61	121	16.9
		Kitise	59	58	117	16.3
2.	Kibwezi East (238, 33.2%)	Masongaleni	60	60	120	16.8
		Mtito Andei	59	59	118	16.5
3.	Kibwezi West (240 33.5%)	Makindu	60	60	120	16.8
		Kikumbulyu North	60	60	120	16.8
**Total**			**358**	**358**	**716**	**100.0**

Key: Freq. = frequency, Percent = percentage

### Number of small ruminants owned and type of ownership

**Fig 3:**
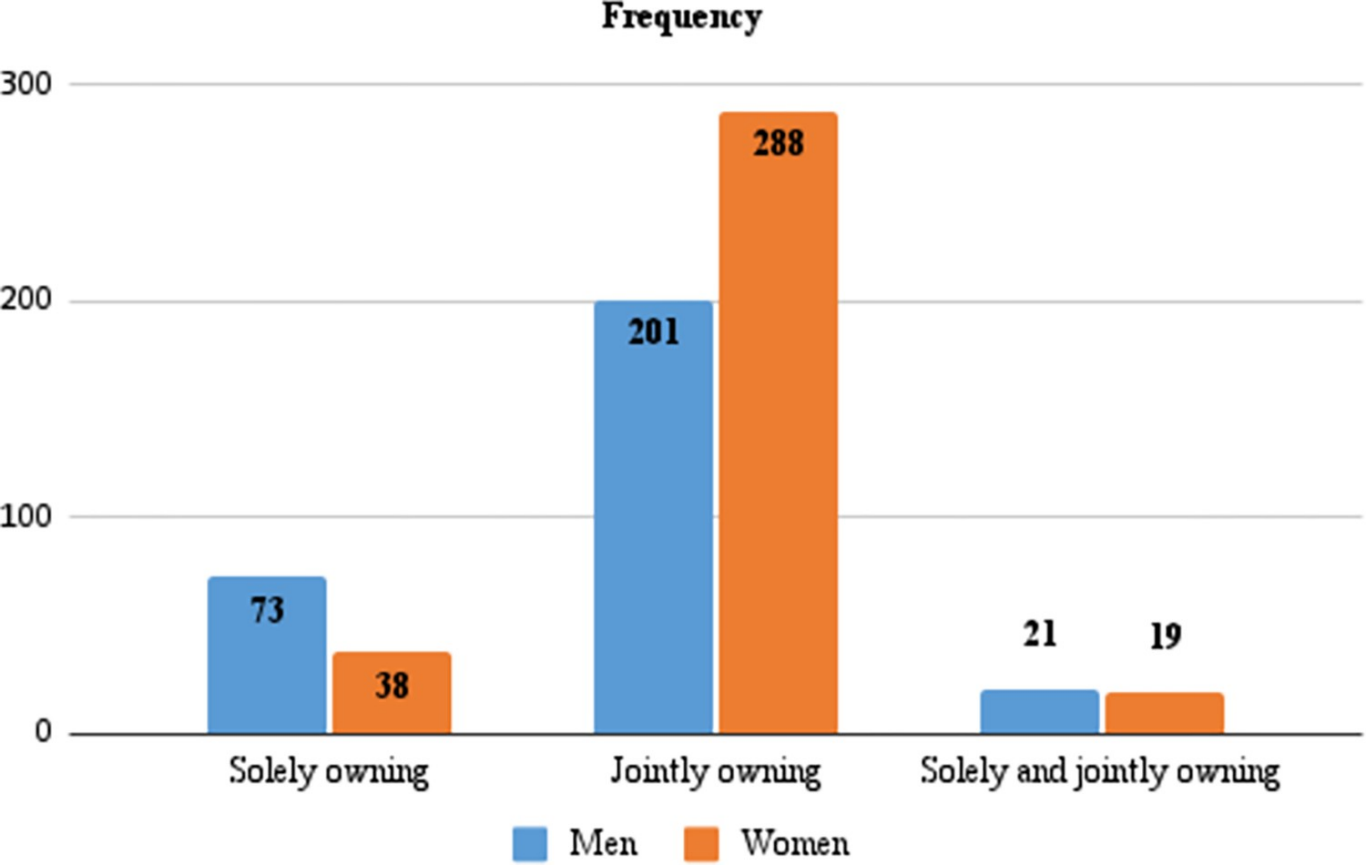
Ownership of small ruminants by gender at the household level.

Households kept on average (28.0±1.18) small ruminants. The average number of small ruminants per household reported by men (29.4±1.20) was higher than that reported by women (26.8±1.65, p>0.05). Overall, the average number of solely owned small ruminants (6.0±0.61) per household was significantly fewer than jointly owned small ruminants (26.6±1.32, p<0.05). The average number of small ruminants solely owned by men (7.91±0.94) was significantly higher (p<0.05) compared to those solely owned by women (4.32±0.79). While sole ownership of small ruminants was reported by more men (24.7%) than women (11.0%), the proportions of men and women that solely owned small ruminants was generally low. In contrast, more women (83.5%) than men (68.1%) reported owning small ruminants jointly with other household members. The average number of jointly owned small ruminants per household as mentioned by men (28.6±2.04) were more compared to those mentioned by women (25.2±1.72). The number of men and women respondents that reported owning small ruminants solely, jointly or both solely and jointly are illustrated in **Fig 3**.

A significant proportion of the farmers co-owned small ruminants with their spouses (87.7%), followed by other household members (35.0%) and with non-household members (0.8%). Slightly more women (88.9%) than men (86.0%) reported co-owning small ruminants with their spouses indicating a strong spousal agreement when it comes to reporting co-ownership, while a moderate proportion of men (37.4%) compared to women (33.2%) reported co-owning small ruminants with other household members. A small proportion of men (1.4%) and women (0.3%) co-owned small ruminants with non-household members.

Joint and sole ownership of small ruminants influenced the level of autonomy men and women had over small ruminants. Regarding solely owned small ruminants, a higher proportion of women than men reported that they could give as gift (52.6%, 15.1%), sell off (71.1%, 19.2%), give as loan (13.2%, 8.2%), pledge as collateral (10.5%, 4.1%), slaughter (78.1%, 15.1%) and look after small ruminants (42.1%, 31.5%), respectively, without consulting their spouses or other household members. Overall, more women (45.6%) than men (15.4%) reported that they could not carry out any of the six activities assessed on solely owned small ruminants without consulting their spouses as presented in Table 2.

**Table 2:** Activities carried out by men and women on solely owned small ruminants without consultation.

Activity	Men		Women		p-value
	Freq.	Percent (%)	Freq.	Percent (%)	
Give as gift	11	15.1	20	52.6	0.004
Sell off	14	19.2	27	71.1	0.000
Give as loan	6	8.2	5	13.2	0.409
Pledge as collateral	3	4.1	4	10.5	0.603
Look after small ruminants	23	31.5	16	42.1	0.001
Slaughter	11	15.1	30	78.1	0.000
Do none of the above	39	15.4	114	45.6	0.000

Key: Freq. = frequency, Percent = percentage. Number and proportion of men and women respondents that reported they could undertake the activities out of those that responded to the question.

Similarly, more women than men reported they could give as a gift (11.4%, 4.1%), sell off (20.8%, 5.4%) and slaughter (31.3%, 26.1%) jointly owned small ruminants, respectively, without consulting their spouses or other household members. However, more men (75.2%) than women (39.7%) reported looking after jointly owned small ruminants without consulting their spouses or other household members. n. A significantly higher proportion of women (41.7%, p<0.05) than men (22.1%) reported that they could not carry out any of the six activities assessed on jointly owned small ruminants without consulting their spouses. There were significant differences in the proportions of men and women who could undertake the assessed activities on jointly owned small ruminants without consultation with spouses or other household members, although the proportions were low indicating that consultation with spouses and other household members happened in most cases as shown in **Table 3**.

**Table 3:** Activities carried out by men and women on jointly owned small ruminants without consultation.

Activity	Men		Women		p-value
	Freq.	Percent (%)	Freq.	Percent (%)	
Give as gift	9	4.1	35	11.4	0.003
Sell off	12	5.4	64	20.8	0.000
Give as loan	6	2.7	6	2.0	0.568
Pledge as collateral	5	2.3	5	1.6	0.603
Look after small ruminants	167	75.2	122	39.7	0.000
Slaughter	28	12.6	96	31.3	0.000
Do none of the above	49	22.1	128	41.7	0.000

Key: Freq. = frequency, Percent = percentage. Number and proportion of men and women respondents that reported they could undertake the activities out of those that responded to the question.

In response to the question (Q. for all small ruminants that you own solely or jointly, what can your spouse do on his/her own, without consulting you?), the majority of men and women farmers expected their spouses to consult them before gifting, selling off, loaning, slaughtering, or pledging as collateral solely or jointly owned small ruminants. However, more women (63.4%) than men (33.9%) had no issue with their spouses looking after solely or jointly owned small ruminants without consulting them. The number and proportions of respondents who reported their spouses can undertake the assessed activities on solely or jointly owned small ruminants without consultation are detailed in **Table 4**.

**Table 4:** Activities that spouses could undertake on solely and jointly owned small ruminants without consultation.

Activity	Men		Women		p-value
	Freq.	Percent (%)	Freq.	Percent (%)	
Give as gift	25	8.5	11	3.2	0.004
Sell off	46	15.6	25	7.2	0.001
Give as loan	20	6.8	2	0.6	0.000
Pledge as collateral	14	4.7	3	0.9	0.002
Look after small ruminants	187	63.4	117	33.9	0.000
Slaughter	59	20.0	28	8.1	0.000
Do none of the above	79	26.8	211	61.2	0.000

Key: Freq. = frequency, Percent = percentage. Number and proportion of men and women respondents that reported their spouses could undertake the activities out of those that responded to the question.

### Selection of livestock species and breeds for rearing

The majority of the households owned small ruminants of indigenous breeds with more women (83.5%) than men (81.8%) reporting that their households owned indigenous breeds at the time of the study. Significantly more women (62.0%, p<0.05) than men (38.0%) considered small ruminants to be the most important livestock species in supporting their households’ livelihoods. On the other hand, more than twice as many men (68.8%) as women (31.2%) considered small ruminants to be the most important livestock species in supporting their livelihood. This finding is intriguing in that while women acknowledge the valuable role played by small ruminants in the household, they do not consider small ruminants as contributing much to supporting their wellbeing. The FGDs sessions revealed that poultry was the most important livestock species in supporting the livelihood of women followed by small ruminants. Ownership of genetically improved small ruminant breeds was reported by 3.1% and 1.0% of men and women, respectively. For respondents that did not own improved small ruminant breeds, a relatively higher proportion of women (20.9%) than men (11.0%) felt that their livelihoods could improve if they kept improved breeds of small ruminants.

### Gender roles and practices around the performance of small ruminants’ husbandry tasks

All the production tasks assessed had both men and women participating to varying degrees. However, more women than men participated in 13 of the tasks assessed in the one-year recall period. More men than women participated in less frequently undertaken activities like disease preventive measures (53.5%, 28.7%), disease curative measures (58.2%, 24.7%), slaughtering (29.4%, 16.5%), breeding (18.8%, 17.6%) and sale of live small ruminants or products from small ruminants (69.6%, 59.2%), respectively. On the other hand, more women than men participated in the frequently undertaken production activities (on daily basis), among the feeding, watering, grazing (browsing), checking the health and sharing workload. **Table 5** details how frequently men and women participated in the 15 assessed tasks on small ruminant production.

**Table 5:** Participation of men and women in different small ruminant production activities.

Activity	Gender	The number of farmers that reported undertaking activity:								Total count (n)	Percent. (%)
		Daily	Per week		Per month		Every three months	Per year			
			Twice	Once	Twice	Once		Twice	Once		
Feeding	Men	59	8	6	4	1	2	0	4	84	24.3
	Women	75	3	12	2	1	0	3	21	117	33.1
Watering	Men	158	23	11	1	5	5	0	13	216	61.0
	Women	203	4	5	0	2	2	0	25	241	68.1
Grazing/ browsing	Men	82	22	32	1	5	1	1	17	161	47.4
	Women	131	18	11	0	4	0	0	32	196	55.7
Checking health	Men	132	18	35	4	7	10	11	17	234	68.8
	Women	196	13	22	2	8	8	1	2	252	71.6
Disease prevention	Men	10	12	26	20	44	41	4	25	182	53.5
	Women	0	9	5	5	21	47	9	6	102	28.7
Curative measures	Men	1	4	2	6	21	36	23	105	198	58.2
	Women	1	9	5	6	17	15	5	29	87	24.7
Milking	Men	19	1	2	0	0	0	0	5	27	7.9
	Women	92	3	3	0	0	1	3	27	129	36.6
Cleaning small ruminant shelters	Men	19	13	35	2	36	22	9	10	146	41.6
	Women	29	30	55	12	32	9	17	43	227	64.5
Slaughter	Men	0	0	0	0	1	6	20	73	100	29.4
	Women	0	0	0	0	1	0	3	54	58	16.5
Breeding	Men	1	0	0	1	3	18	17	24	64	18.8
	Women	1	3	0	8	19	3	7	21	62	17.6
Sire services	Men	0	0	0	1	1	5	23	38	68	20.0
	Women	0	0	1	6	10	3	9	21	50	14.2
Selling live small ruminants or products	Men	1	0	1	3	3	14	24	57	103	69.6
	Women	1	0	4	13	3	8	24	89	142	59.2
Breed selection	Men	0	1	0	2	2	7	22	44	79	23.2
	Women	0	0	0	4	19	6	14	44	87	24.7
Sharing workload	Men	63	16	20	0	2	1	5	6	113	33.2
	Women	157	3	7	1	8	0	0	10	186	52.8
Setting aside small ruminants’ products for home consumption	Men	5	2	2	2	3	1	1	12	28	8.2
	Women	18	4	2	4	21	4	21	41	115	37.7

With regards to the amount of time spent on each production task per week, women spent significantly (p<0.05) more hours than men in tasks like feeding, sire selection, disease prevention and taking curative measures on small ruminants. Overall, women spent on average more hours per week than men carrying out 10 out of the 15 tasks assessed. Whereas more women than men participated in sharing workload in the household, watering, milking, setting aside products for home consumption and selecting breeds and species to rear, men spent more hours per week compared to women undertaking these tasks as detailed in Table 6.

**Table 6:** Time (hours) spent per week by men and women performing different production tasks.

No.	Small ruminant production tasks	The average amount of time (hours per week) men and women spent carrying out the tasks (Mean±SD)		p-value
		Men	Women	
1.	Feeding	2.54±2.72	4.28±5.19	0.01
2.	Providing drinking watering	0.52±0.79	0.41±1.08	0.23
3.	Grazing/browsing	3.99±2.26	4.06±2.98	0.09
4.	Checking health	0.29±0.54	0.34±1.34	0.59
5.	Disease prevention measures	0.56±0.70	1.05±1.42	0.00
6.	Curative/treatment measures	0.66±1.02	1.40±1.67	0.00
7.	Milking	0.48±0.51	0.03±0.17	0.00
8.	Cleaning small ruminant shelters	1.13±1.57	1.14±1.41	0.96
9.	Slaughtering	0.94±0.72	1.10±1.04	0.25
10.	Breeding	3.88±4.75	3.11±4.32	0.35
11.	Sire selection	1.01±1.60	3.14±4.45	0.00
12.	Setting aside products from small ruminants for home consumption	0.64±0.62	0.51±0.93	0.49
13.	Marketing live animals or products from small ruminants	1.58±1.44	1.97±2.35	0.15
14.	Selecting breed to keep	1.36±2.32	1.30±1.81	0.85
15.	Sharing workload among household members	2.00±3.02	1.11±2.60	0.01

Key: SD-standard deviation

Qualitative data from FGDs revealed that women were mainly involved in cleaning small ruminant housing structures, constructing housing structures, taking sheep and goats for grazing [browsing], confining small ruminants at night, and releasing them in the morning as illustrated in excerpts from both the men and women FGDs:

"*Making sure that they [sheep and goats] sleep in a clean environment…Releasing the goats first thing in the morning*…*Constructing a goat house*" (Women FGD-p5)

‘*It’s the woman who is the person to take them grazing or the one who is around [home]"*

(Men FGD)

The men were mainly involved in the treatment and vaccination of small ruminants including buying medication for the sick animals and vaccines as illuminated in the following excerpt:

[Question: who purchases drugs, food and vaccines for the sheep and goats?]

“*That is done more by the men*" (Women FGD)

Occasionally the men helped women in carrying out other roles like grazing and confining small ruminants:

"*Goats are grazed and brought back from the grazing fields by women although they are also assisted by men*." (Women FGD)

### Intra-household decision-making around production and income

Responding to the question, “Q. When decisions are made regarding the production tasks assessed, who is it that normally makes the decision?”, the majority of men and women reported the male household head as responsible for making decisions on most of the production tasks. However, there was spousal disagreement regarding who mostly makes decisions on 5 of the 15 tasks assessed. The majority of men reported that their spouses were the decision-makers on milking while assigning themselves the role of decision-making on the other 14 tasks. More women respondents considered themselves decision-makers in tasks like disease prevention, taking curative measures, slaughtering, and marketing live small ruminants or products from small ruminants. Overall, however, the majority of women respondents reported that men were the decision-makers in 11 out of 15 assessed activities as presented in Table 7.

**Table 7:** Person responsible for decision making on different production tasks.

Production task	Men			Women		
	Male HH head	Spouse	Other HH member	Male HH head	Spouse	Other HH member
Feeding	73	65	3	97	84	4
Watering	181	147	22	219	154	19
Grazing	143	103	17	177	137	16
Checking health	220	137	12	233	187	7
Disease prevention	173	100	15	77	81	0
Curative measures	190	101	13	66	67	2
Milking	18	22	1	128	29	5
Cleaning	120	86	12	208	120	22
Slaughter	93	73	7	39	50	0
Breeding	61	44	6	51	45	0
Sire services	59	32	1	39	37	0
Set aside for home consumption	25	24	0	112	71	0
Market live animal	87	73	2	112	121	3
Breed and species selection	76	49	4	71	69	1
Sharing workload among household members	97	95	16	176	117	18

Key: HH-household. Numbers refer to the frequencies of men and women respondents who reported male HH, spouse or another HH member as the person who usually makes the decision on the assessed production task

The farmers were asked to gauge their contribution to decisions on small ruminant production activities on three levels; little to no input into decisions, input into some decisions, and input into most or all decisions. More men (61.7%) than women (43.2%) reported inputting into most or all decisions regarding the choice to rear small ruminants (p<0.05). Comparable results were recorded on input into decisions on the use of income generated from the sale of live small ruminants where more men (50.2%) than women (49.1%) inputted into most or all decisions. In contrast, significantly more women (57.9%) than men (40.7%) contributed to most or all decisions regarding the amount of outputs from small ruminants to set aside for home consumption rather than selling (p<0.05). **Table 8** summarizes the number of men and women who took part in decision-making on various production activities.

**Table 8:** Men and women who took part in decision-making on various production activities.

Activity	Little to no input in decision making		Input in some decisions		Input into most or all decisions		P-value
	Men	Women	Men	Women	Men	Women	
Input into the decision to rear small ruminant	8	39	92	104	161	115	0.000
Input into the decision on the use of income from sale of small ruminants of small ruminant products	28	38	111	104	140	137	0.771
Input into the decision on the amount of outputs from small ruminants to set aside for home consumption	44	34	106	86	103	165	0.000
Input into the decision on small ruminants feeding	1	2	17	36	59	32	0.002
Input into the decision on small ruminants watering	5	2	73	38	115	110	0.001
Input into the decision on cleaning small ruminants structures	3	3	22	27	101	92	0.739
Input into the decision on the sale of small ruminants & its products	0	6	31	35	66	55	0.000

The numbers refer to the frequency of men and women respondents who reported to have taken part in the decision making around small ruminant production activities assessed. An assumption was made that respondents who answered the questions had such decisions to be made and therefore partook in them.

To assess the extent respondents could participate in decision-making around small ruminant production activities if they wanted to (both farmers that took part and those that did not take part in decision-making), more men (51.3%) than women (35.5%) reported they could participate to a high extent in decisions on whether to rear small ruminants or not if they wanted to. Similarly, a higher proportion of men (17.2%) than women (12.5%) felt to a high extent that they could access the necessary information on small ruminants raising if they wanted to as presented in **Table 9**.

**Table 9:** Extent of participation in decision making on small ruminants’ production activities by men and women if they wanted.

Activity	Not at all		Small extent		Medium extent		High extent		P-value
	Men	Women	Men	Women	Men	Women	Men	Women	
Make decisions on small ruminants	7	7	20	43	101	119	135	93	0.001
Access information on small ruminants	80	69	96	54	74	142	52	38	0.000
Small ruminant feeding	0	0	8	14	25	34	51	69	0.002
Decisions on watering	1	0	47	15	63	51	105	175	0.000
Decisions on cleaning animal structures	0	0	20	13	36	54	90	160	0.000
Amount of output to set aside for home consumption	0	0	4	4	12	45	12	66	0.000
Sale of small ruminants or products from small ruminants	1	4	8	24	33	42	61	72	0.005

The numbers refer to the frequency of men and women respondents who reported they could partake in decision-making on assessed small ruminant production activities if they wanted to.

Whereas sole ownership of small ruminants by women was relatively higher than men, from the emic perspectives captured in the FGDs, men were exhibited more agency in decision-making even when the small ruminants belonged to the women as illustrated in the excerpts from men and women FGDs:

*’Similarly*, *chickens belong to women and in case I am in need of chicken meat*, *I will request the woman to slaughter a chicken for me*, *but goats and sheep belong to me*." (Men FGD)

*"You know goat is much bigger compared to chicken and therefore*, *the man is expected to gauge whether the current need of the family is much bigger to be solved by selling a chicken*, *or if it requires selling of a goat*" (Women FGD)

From the qualitative data it was evident that in the study setting men were the de facto owners of small ruminants and had a higher power position in the important decisions touching on small ruminant production and management. The men were seen as key decision-makers in aspects of small ruminants which were perceived to be more important. In the study setting, decisions that had financial implications on the household and those that touched on the welfare of the livestock were regarded as important; for example, the sale of small ruminants or products from small ruminants, vaccination, and treatment of sick small ruminants. Both men and women were of similar view as illustrated in the excerpt:

"*So for sheep and goats*, *the man says whether it will be vaccinated or not”* (Women FGD)

### Perceptions on the role and value of small ruminants in the community

In the study setting, characteristic of a rural smallholder farming community with semi-arid limited arable land for crop farming, small ruminants were reared mainly for economic empowerment, household subsistence and consumption inform of meat and milk as seen in the following excerpts:

"*We keep goats for milk production*, *for meat and when in need of money*, *one can sell goats*." (Women FGD)

"*Apart from money*, *milk and meat*, *goats and sheep are a source of manure which we use in crop farming*." (Men FGD-p3)

Sheep and goats were also seen as financial security that could easily be disposed-off in exchange for money to meet important family needs and at the same time safeguard large livestock like cattle as illustrated in the excerpts from men and women FGDs:

"*Goats act as security for cattle*. *When the money needed can be gotten from the sale of a goat*, *then there is no need to sell a cow*." (Men FGD)

"*Yes*, *a child may be sent home from school for fees; you may sell goats and sheep*, *get money and take the child back to school*." (Women FGD)

There was a cultural value attached to the rearing of small ruminants by especially men. The small ruminants were seen as important for kinship as they were used for paying the bride price when marriages were being formalised as in the excerpt:

"*They help us very much when it comes to bride price payment in the in-law’s family*. *Culturally we use goats for dowry payment*." (Men FGD-p6)

In conclusion, while women in the present study felt small ruminants were important livestock species in supporting their household’s livelihoods, they also recognized that small ruminants were important species in supporting their livelihoods after poultry. This should be taken into consideration by intervention projects targeting to empower women through small ruminant production as it may inform the project implementation. The study has also identified small ruminant production facets that can be targeted for the empowerment of women smallholder farmers. One of the positive results of the present study is the high number of women who owned small ruminants either solely or jointly at the household level. Another favourable result was that women were still able to benefit from small ruminants even where ownership of the animals was solely by their husbands. While ownership of small ruminant is important and should be encouraged, in households where ownership of livestock assets by women seem not to be feasible, efforts should be directed at enabling women to benefit from the household livestock assets without necessarily having to own them. Women performed most of the production tasks around small ruminant production and made decisions without financial implications while the men appeared to be the de facto owners of small ruminants with a higher power position in production decisions with financial implications. Intervention projects should put measures to correct these gender biases when implementing their programs, particularly those seeking to empower women through small ruminant production.

## Discussion

Small ruminant production is an important activity that complements other income-generating activities in the household. Women in the present study not only performed most of the tasks around small ruminant production but also owned more sheep and goats compared to men. However, perception of ownership appeared to vary between men and women with both genders owning and having varying levels of control over small ruminants. In Kenya, small ruminants are largely considered women’s “property”, although the notion of “ownership” appears to be complex, context-specific, and highly dynamic [49]. The findings are consistent with other studies that attributed greater ownership of small ruminants to women, although with varying degrees of authority over decision-making [23, 36, 37, 50–53]. For instance, in Gambia, Jaitner *et al*. [36] established that goats were non-pooled household assets independently owned and managed by household members, who were more often than not women. Similarly, more women than men owned goats and made decisions on their management in parts of Kenya and Uganda [37]. Contrary findings with regards to goat ownership have also been reported. Semakula *et al*. [54] for instance highlighted the predominance of men in goat ownership and decision-making, while Byaruhanga *et al*. [55] reported more men than women as sole owners of goats. Ownership of small ruminants by men and women is influenced by the level of commercialization of small ruminant production enterprises as men usually take over control following increased intensification [56], while the women are mostly in charge when production is mainly subsistence.

Joint ownership of small ruminants between the husband and wife in the present study was predominantly reported by men compared to women. This was in sharp contrast to a study by Tavenner *et al*. [35] on dairy cattle where joint ownership between the husband and wife was reported by more women than men. It is either there is a tendency of each gender to claim ownership of livestock species belonging to their spouses and to claim a stake in areas where they are disadvantaged when reporting or they are just unable to accurately assign ownership. Inability to correctly assign ownership of livestock assets has previously been attributed to two reasons; either to the inability of the reporting household member to know the correct numbers owing to multiple gender-defined activities contributing to household income that make it difficult for a household member to accurately keep abreast of the numbers [57] or due to the “telescoping” problem that was expounded by Deaton & Grosh [58]. Ownership patterns of small ruminants vary widely between regions, communities, and depending on the gender of the person interviewed. The present study established that ownership of small ruminants by different household members has a bearing on what they can do and decisions they can make on different aspects of sheep and goat production. Women appear seemed to be overstrained with most of the production and management tasks around small ruminant production. Encouraging fair distribution of workload and labour among different household members is therefore one area that development programs working towards women empowerment can target to effect change. The involvement of women as sole and joint owners of small ruminants underscores the importance of targeting men and women in intervention programs for small ruminants [55]. While the women owned higher numbers of small ruminants in the present study, our results show that ownership alone does not confer control or guarantee decision-making rights to the women especially when such decisions have financial implications. More men than women made decisions on the use of income from small ruminant production, whereas more women than men contributed to decisions regarding the amount of proceeds from small ruminant production to set aside for home consumption. There was a unanimous agreement in both men-only and women-only FGDs that decisions regarding when to sell, when to treat, and when to vaccinate small ruminants were mostly made by men. Literature abounds with similar findings where women offer most of the production labour while the men enjoy greater control over the finances and decision-making [25, 59, 60]. This type of disparity derails women’s empowerment efforts and should be targeted by intervention programs that seek to empower women through livestock production. Women can only attain self-efficacy if they are supported to not only own small ruminants but also have control over financial and decision-making rights. Historical and structural gender inequalities that are biased against women when it comes to access to information have been identified as some of the factors hindering women from attaining self-actualization [38, 59]. Thus, eliminating existing socio-cultural norms and practices that limit women’s access to information and training is one way of establishing a fair balance [49]. In most parts of SSA, the prevailing norms view women as providers of “unpaid family labour" which leaves them with little or no time to attend training [44]. This is exacerbated by the failure of the training organizers to take into consideration the poor mobility and heavy workload of women when identifying venues and setting times for the training. It has been established that more women attend training sessions when they are held on weekends, during off-peak hours and when such training takes a shorter time [51]. These aspects should be considered by intervention programs seeking to use training as part of the methods of empowering smallholder women farmers.

The women’s tendency to over-report joint decision-making with their spouses in domains where they are disadvantaged like the sale of livestock assets, access to and use of income has been described [61]. Since ownership patterns of small ruminants tend to change over time [49], intervention programs that seek to impact women’s livelihoods should implement measures that safeguard already achieved milestones as well as increase women’s access to and use of income from livestock production. Efforts should be directed towards ensuring women are not just ‘owners’ of small ruminants but also decision-makers on household livestock assets.

The present study has revealed that few women had control over small ruminants and the leeway to use the animals for various purposes including gifting and slaughtering without consulting their spouses even when the animals were solely owned by their husbands. While this is positive, it is an indicative reason why a broad approach should be adopted when it comes to women’s empowerment through livestock production. The focus should not be limited to women’s ownership of livestock assets alone, since there are instances where women still benefit to a large extent from the livestock assets like milk and income from the sale of livestock despite not owning them [62]. Emphasis should be put on women’s ability to access income and derive benefits from the household’s livestock assets rather than ownership. For projects aiming to support women to own small ruminants, then control and decision-making rights should be considered alongside ownership, use and access rights [63]. Whereas supporting the women to own small ruminants is a good approach to uplifting women, studies have cautioned that focusing on ownership alone can elicit opposite results and decrease women’s bargaining power if decisions, particularly those with financial implications remain in the hands of men [23, 64], as was the case in the present study. In such a case, therefore, appropriate measures should be instituted that improve women’s bargaining power with regard to access, use and control over livestock assets. Women’s ownership of small ruminants should also be safeguarded especially when the enterprises become more profitable because this is the time men tend to take over control [60]. Should this trend persist, women will continue to bear the burden of performing most of small ruminant production tasks as the men enjoy financial proceeds [42, 43]. There were discernible variations in intra-household reporting between men and women on ownership and control of livestock assets. The figures reported by each gender should be treated with caution bearing in mind that men may not keep abreast with the number of animals owned since they are rarely involved in daily management of small ruminants. The many responsibilities around small ruminant management carried out by women may also give them a false impression of control over the livestock assets even when men are in charge.

In conclusion, potential exist for empowering women through small ruminant production if factors that promote women productivity are identified and strengthened while those that hinder their productivity are documented and rectified. Whereas progress has been made to shrink the gender disparity around decision-making, labour allocation, control and ownership of livestock assets, the present study identified facets where women are still disadvantaged that development programs should target to correct through community specific interventions and government policies. The study offers three implications on the design of livestock interventions to empower women, the interventions should ensure that; 1) women are not just owners of livestock assets but also share power and decision-making rights in all aspects of production, 2) production labour is shared equitably between men and women and, 3) women access benefits from livestock production even when animals are owned by men.

## Supporting information

S1 Appendix WELI instructional guide detailing definitions of the task assessed in the study. (PDF)

S2 Appendix The complete WELI quantitative questionnaire used in the study (source: International Livestock Research Institute in accordance with its creative commons license). (PDF)
