# Supplementary material for: Women’s empowerment and intra-household gender dynamics and practices around sheep and goat production in South East Kenya
Source: PLoS One. 2022 Aug 4;17(8):e0269243. doi: 10.1371/journal.pone.0269243 (PMC9352016; doi:10.1371/journal.pone.0269243)
Supplement: S1 Appendix — (PDF) [file pone.0269243.s001.pdf]

WELI INSTRUCTIONAL GUIDE

For WELI questionnaire added to Pro WEAI Livestock enhanced Module

Table of Contents

1. Introduction 2
2. Preparing the data collection devices and conducting the survey on digital devices 3
3. General Instructions for All Sections 5
4. Conversion Factors 5
5. UNIVERSAL CODES: 6
6. Instructions by Section: Household Questionnaire 6

I. Household Structure and Choice of Respondents 6
II. Informed consent 7
III. MODULE G1. INDIVIDUAL IDENTIFICATION 7
IV. MODULE B. HOUSEHOLD LISTING AND DEMOGRAPHICS (REQUIRED). 8
V. MODULE G2: ROLE IN HOUSEHOLD DECISION-MAKING AROUND PRODUCTION AND INCOME 8
Table G2.01 – G2.07 8
Items in Table G2.01 – G2.07 11
VI. MODULE G2: ROLE IN HOUSEHOLD DECISION-MAKING AROUND PRODUCTION AND INCOME 12
Table G2.09 – G2.20 12
Definition of items in Table G2.08 – G2.20 12
List of activities in Table G2.12 – G2.20 13
VII. MODULE G3(A): ACCESS TO PRODUCTIVE CAPITAL 15
Questions G3.01 to G3.06 15
Table G3.06 - G3.11 16
Definition of items in Table G3.06 - G3.11 16
VIII. MODULE G3 (B): Access to credit 17
IX. MODULE G4: Time allocation 18
Determining Activities ✔ 19
X. MODULE G5: Group membership 22
XI. MODULE G6: Physical mobility 22
XII. MODULE G7: Intrahousehold relationships 22
XIII. MODULE G8(A): Autonomy in decision making 23
XIV. MODULE G8(B): NEW General self-efficacy scale (OPTIONAL) 23
XV. MODULE G8(C): Life satisfaction (OPTIONAL) 23
XVI. End of the interview 24

1. Introduction

Developed by a multidisciplinary team of researchers from the International Livestock Research Institute (ILRI) and Emory University, the WELI module is a new standardized tool measure the empowerment of women involved in the livestock sector.

Note to survey designers: The information in module G1 can be captured in different ways; however there must be a way to: (a) identify the proper individual within the household to be asked the survey, (b) link this individual from the module to the household roster, (c) code the outcome of the interview, especially if the individual is not available, to distinguish this from missing data, and (d) record who else in the household was present during the interview. This instrument must be adapted for country context including adding relevant examples and translations into local languages when appropriate.

Note to enumerators: This questionnaire should be administered separately to the primary and secondary respondents identified in the household roster of the household level questionnaire. You should complete the coversheet for each individual identified in the "selection section" even if the individual is not available to be interviewed for reporting purposes. For some surveys (such as those focusing on nutrition outcomes), the female respondent may be the beneficiary woman or mother or primary caregiver of the index child (also the respondent for the pro-WEAI nutrition module). Please make sure that she is also the person interviewed for this questionnaire and that the male respondent is her spouse/partner (if applicable). Please double-check to ensure:

- You have completed the roster section of the household questionnaire to identify the correct primary and/or secondary respondent(s);
- You have noted the household ID and individual ID correctly for the person you are about to interview;
- You have gained informed consent from the individual in the household questionnaire;
- You have sought to interview the individual in private or where other members of the household cannot overhear or contribute answers

2. Preparing the data collection devices and conducting the survey on digital devices

a. Download and install ODK collect from app store to your Android mobile device.
b. Launch the app and on the top right side see these three dots.

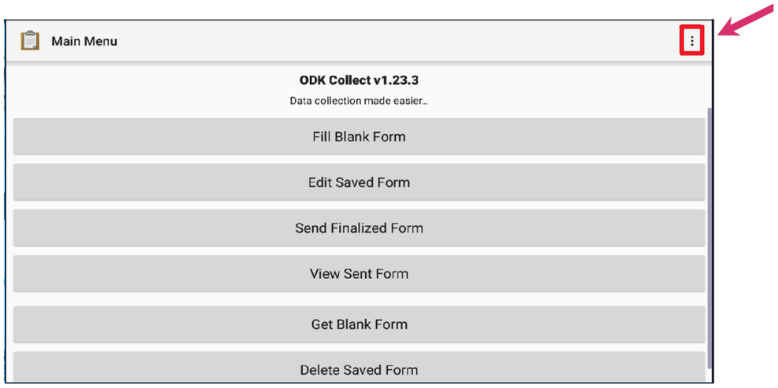

c. Click on the three dots; a drop down will open.

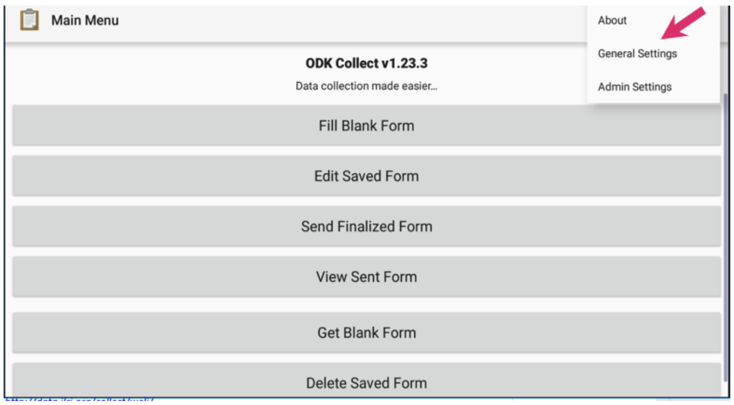

d. Click on general setting then enter the following as your server option.
URL: *http://data.ilri.org/collect/weli/*
username: *weli*
Password: *Yhvn42CHbW*

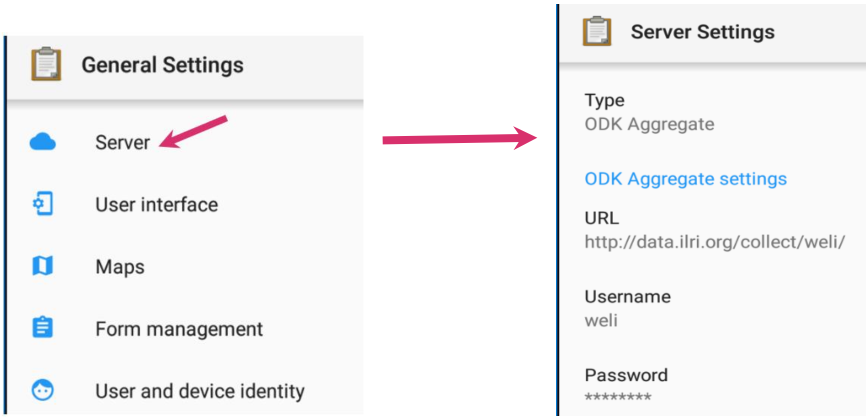

e. Then go back to the main ODK display and click on 'Get blank form'

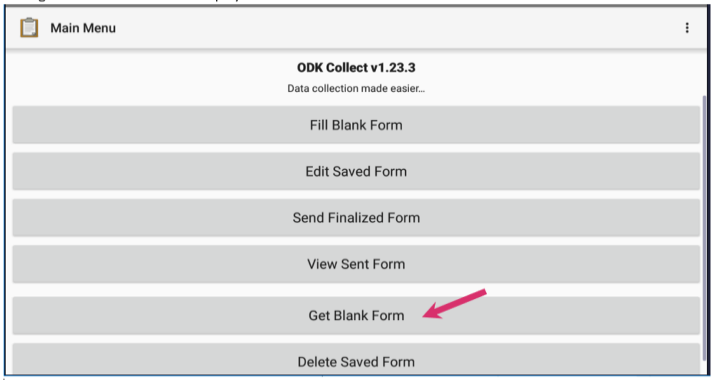

f. Select *weli_ilri_2019_KE* as the most updated tool and 'Get selected'

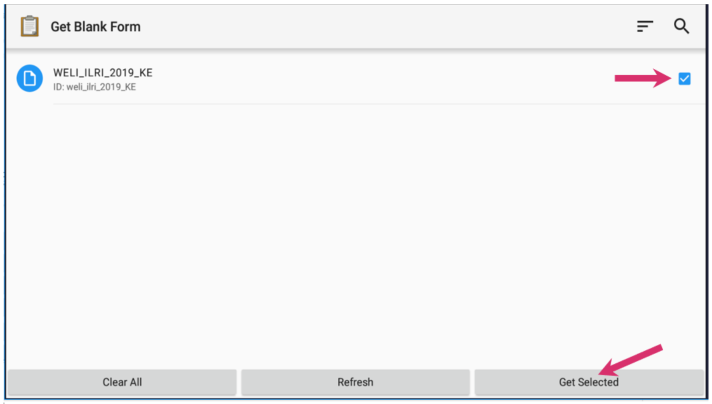

g. Click OK to download the form

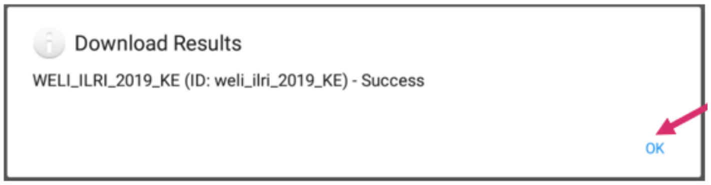

h. Once this is downloaded, click on 'Fill blank form' to launch the data collection app.

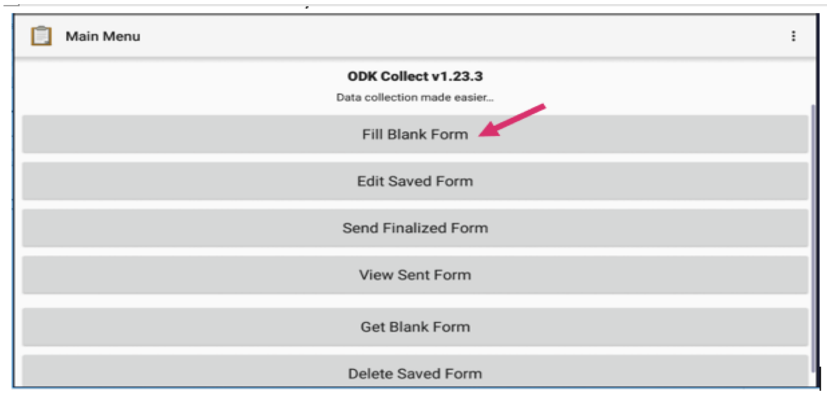

3. General Instructions for All Sections

- Do not read the respondents the list of coded answers unless explicitly instructed to "READ." Instead, let the respondent give you their response to the question and them select the most appropriate response from the list of the coded answers. If the respondents answer is unclear, it might mean that the respondent did not understand the question so probe further and rephrase the question for the respondent to understand.
- Month should always be written in numbers from 1-12.
- Year should always be written in 4 digits.
- Complete dates such as birth dates or interview dates should have the following format: DD-MM-YYYY (i.e. 2-digit day, 2-digit numeric month, 4-digit year)
- Percentages should always be written from 0-100.
- Start with the household questionnaire, and then the individual questionnaires. If possible, and as facilitated by field team composition, male interviewers should interview the primary male respondent and female interviewers the primary female respondent.
- It is ok for other household members to assist with the recall for questions asked to the household head or spouse as part of the household-level questionnaire ONLY.
- For the individual questionnaire, if possible, individual respondents should be interviewed separately, without other household members or outsiders present. It is NOT advisable for other household members to assist with recall for questions on the individual questionnaire.
- While the research will estimate different indicators, the main one being the Women Empowerment in Livestock Index (WELI), the mentioning of the research being conducted should focus on the project under which the WELI estimates are needed either for monitoring project progress or informing project activities, and not necessarily describing the tools of analysis (i.e. WELI etc). As indicated in the study consent form, the questions in the study directly focus on decision making within the household. In order not to create the impression that we want to change or influence gender roles or women empowerment using the survey, which might skew or bias results, it is best to introduce the project in question and what the tool covers in terms of household decision making, and not analysis of the data (women empowerment).

4. Conversion Factors

- 1 kilometer = 0.6 miles
- 1.67 kilometers = 1 mile
- 1 mile = 1667 meters
- 1 FOOT = 0.30 METRES
- 1 YARD = 0.91 METRES
- 1 KILOMETRE = 0.62 MILES
- 1 acre=100 decimals
- 1 hectare = 2.47 acres
- 10000 meters sq. = 1 hectare
- 4048 meters sq. = 1 acre
- 1 meter sq. = 0.000245 acres

5. UNIVERSAL CODES:

The following codes may be appropriate in any question in the household survey. In no case (with the exception of skip patterns) should a question be left blank. If you are unsure, make a note and ask your supervisor at the end of the questionnaire how to fill the particular question.

Don't know………………………………………………88

Not applicable (N/A)/No decision made........98

Other specify …………………………………………… 99

6. Instructions by Section: Household Questionnaire

I. Household Structure and Choice of Respondents

A clear and standardized definition of the household is important since different household definitions result in different household compositions, and can have significant impacts on variation of outcome indicators particularly surrounding labor and consumption (Beaman and Dillon 2012).

To facilitate cross-country comparisons, we recommend the standard definitions to be used to identify who qualifies as a "household", and who qualifies as an interview subject, or a "primary" and "secondary" respondent.

Several multi-purpose household surveys define a household as a group of people who live together and take food from the "same pot" (Ayad et. al., 1994; Glewwe, 2000). The important part of this definition is that the group of individuals shares at least some common resources and makes some common budget and expenditure decisions. A household member is someone who has lived in the household at least six (6) months, and at least half of the week in each week in those months. Even those persons who are not blood relations (such as servants, lodgers, or agricultural laborers) are members of the household if they meet these qualifications, and alternatively, individuals who sleep in the household, but do not bear any costs for food or do not take food from the same pot, are not considered household members. This definition, including more specific examples and guidelines, is embedded in the questionnaires.

Note that in exception to the above rules, the following should be considered as household member:

- A newborn child less than 3 months old.
- Someone who has joined the household through marriage less than 3 months ago.
- Servants, lodgers, and agricultural laborers currently in the household and will be staying in the household for a longer period but arrived less than 3 months ago.

Do not consider as household member:

- A person who died very recently though stayed more than 3 months in last 6 months.
- Someone who has left the household through marriage less than 3 months ago.
- Servants, lodgers, and agricultural laborers who stayed more than 3 months in last 6 months but left permanently.

Good Practice Tips

- If the standard household definition does not make sense in the context where the surveys will be implemented, it is most important is to ensure that enumerators have the same understanding of definitions so that implementation is consistent across households. When in doubt of who to include or exclude, please discuss with your supervisor.

The **primary and secondary respondents** are those who are self-identified as the primary members responsible for decision making, both social and economic, within the household. They are usually husband and wife; however, they can also be other members as long as there is one male and one female aged 18 and over. For example, one might find a widowed mother and her adult son as the primary person responsible for decision making in the household. The index woman can either be the primary respondent or the secondary respondent.

II. Informed consent

Before beginning the interview, it is necessary to introduce the household to the survey and obtain their consent to participate. Make it clear to them that their participation in the survey is voluntary.

After reading out the informed and seeking consent from the respondents, ensure that you sign the duplicate copies of the informed consent for each household and leave one copy with the household after the interview.

III. MODULE G1. INDIVIDUAL IDENTIFICATION

Coversheet: Please make sure that information collected on the coversheet of the individual questionnaire is consistent with information of the coversheet for the household questionnaire. **Having the correct household ID and individual ID (get it from the household roster) is very important.**

G1.01. Household identification – this is a unique identifier assigned to each household. It can either be numeric or alpha numeric. Before heading to any household, ensure that your supervisor has assigned the household ID for the household that you are going to interview. Note down the assigned household ID for ease of reference once you start the interview

- **Tip for survey supervisors:** The best Household ID's to use is "intelligent Household ID" that give more information than just a number. These are best given in alpha-numeric form where for instance, the first letter(s) represent the country, the second letter(s) represent the district/county/woreda etc, and then followed by numeric digit(s) for each household. For example, households interviewed in Nairobi county (Kenya) could be assigned household ID's as KENAI001 … KENAI300 (KE=Kenya, NAI=Nairobi, 001=first household to be interviewed and 300= the 300th household to be interviewed).
- **Tip for enumerators and supervisors:** Please double check that the ID for the household is correct (from sampling list).

G1.02. Name of respondent currently being interviewed – This is to specifically identify the person being interviewed by name. (code from roster in Section B): Provide the name in the following order: Surname, First name. The surname is the same as what is called the "Last name".

G1.03. Sex of respondent - The sex of the respondent is to be given as either **Male** or **Female**.
Good practice: Do not ask this question if the sex is obvious, either by name or by physical observation, however, ask it when in doubt and the member is not present for you to observe.

G1.03a. Who are you interviewing? – This is to specifically identify the person being interviewed, whether it is the primary or the secondary respondent

IV. MODULE B. HOUSEHOLD LISTING AND DEMOGRAPHICS (REQUIRED).

Enumerator: Ask these questions about all household members
Please introduce the section by telling the respondent that to begin the discussion, you would like to talk know a little about each member of your household i.e. their names, their relationship to the primary respondent, and their date of birth. Please list the names of everyone considered to be a member of this household, starting with the primary respondent.

B01: Name of household member – ask for the names of each of the household members, starting with primary respondent, followed by the secondary respondent, and other members in descending order of age. Provide the name in the following order: Surname, First name. The surname is the same as what is called the “Last name”.

B02: What is [NAME’s] sex? – The sex of each member is to be given as either **Male** or **Female**. **Good practice**: Do not ask this question if the sex is obvious, either by name or by physical observation, however, ask it when in doubt and the member is not present for you to observe.

B03: What is [NAME’s] relationship to the primary respondent? – Here you ask, for each member, how they are related to the primary respondent, and not necessarily the household head.

B04: When was [NAME’s] born? (YYYY years) – The best approach is to get the age of a household member is to ask when (year), the household member was born.

V. MODULE G2: ROLE IN HOUSEHOLD DECISION-MAKING AROUND PRODUCTION AND INCOME

Table G2.01 – G2.07

The purpose of this module is to get an idea about men’s and women’s relative roles in decision-making around income-generating activities. Do not attempt to ensure that responses are the same between the male and female respondent. It is okay for them to be different.

When we ask who makes a decision we are now asking for ID codes so we can link decisions to individual respondents Survey designers should insert additional, local examples of activities where relevant.

Please introduce the section by telling the respondent that you would like to talk about ask you some questions about your participation in certain types of work activities and on making decisions on various aspects of household life.

This module includes the following questions:

- **G2.01**: Did you (NAME) participate in [ACTIVITY] in the past 12 months (that is within the last [one/two] cropping seasons), from [PRESENT MONTH] last year to [PRESENT MONTH] this year? This question is answerable by YES or NO. Please note the following:
    - The reference time frame for this question is always 12 months. In some cases, for crop production related activities, it will be helpful to ask the respondent to think about the last two cropping seasons if the area has a bi-annual crop season. In other cases, the reference period should only be one cropping season depending on the number of cropping seasons per year, the intervention and the timing between surveys. This should be decided upon and standardized during the training period.
    - In some languages there is a singular you and a plural you. This question refers to the singular you (the person being interviewed, not the respondent together with his or her family). If the local language does not distinguish between singular or plural, make sure the respondent understands that this applies to just him/her (This will also apply to all other places where we ask about "you" in the individual questionnaire).
    - If the respondent answers "no" he/she did not participate for the activity, then skip to the next activity.
- **G2.02**: When decisions are made regarding [ACTIVITY], who is it that normally takes the decision?
    - IMPORTANT: When you ask who makes a decision indicate the household member ID codes in order to link decisions to individual respondents. You may enter up to 3 member ID codes.
    - If the respondent mentions decisionmakers who are not part of the household, select the following options, whichever is appropriate as indicated by the respondent:
      NON-HH MEMBER (MALE)
      NON-HH MEMBER (FEMALE)
    - If no decisions were made regarding the [ACTIVITY], select NO DECISION MADE and skip to the next activity.
    - If there are more than 3 decisionmakers, ask the respondent to mention the 3 most important ones.
    - Note: if the respondent answers "self" only (i.e. there are no other decisionmakers mentioned except the respondent herself/himself), then skip to question **G2.05**. Questions **G2.03** and **G2.04** ask about the level of input and control the respondent feels he/she has over the activity, but if she/he indicates that he/she alone makes the decision, then we can safely assume they have high input and control over the decision.
    - It is optional to add a question here asking who the respondent would like to make the decision.
- **G2.03:** How much input did you (singular) have in making decisions about [ACTIVITY]?
    - Enter the appropriate response code from the codes listed at the bottom of the module (write one):
      Little to no input in decisions…………………..…..01
      Input into some decisions………..………………..…02
      Input into most or all decisions…………………..…03

Not applicable/No decision made…….98 → If this code is selected then skip to the next activity.

- In some cases in the local language **G2.03** needs to be asked in two separate questions (first ask if any input is made and then ask the level of input).

- **G2.04**: To what extent do you feel you can participate in decisions regarding this [ACTIVITY] if you want(ed) to?
  - Circle one response only from the following categories:
    Not at all…………………….……01
    Small extent………..……………02
    Medium extent…………………….03
    To a high extent…………………..04
  - Even if a respondent did not participate in decisions, he or she may be doing so by choice, like when a decision is delegated to others, or if the respondent has no interest in the particular activity or decision. This question is intended to capture whether the respondent can participate in the decision-making process if they choose to.

- **G2.05**: To what extent are you able to access information that you feel is important for making informed decisions regarding [ACTIVITY]?
  - Circle one response only from the following categories:
    Not at all…………………….……01
    Small extent………..……………02
    Medium extent…………………….03
    To a high extent…………………..04
- **G2.06:** How much input did you have in decisions about how much of the outputs of [ACTIVITY] to keep for consumption at home rather than selling?

Enter the appropriate response code from the codes listed at the bottom of the module (write one):
Little to no input in decisions ……….............01
Input into some decisions………..……………..02
Input into most or all decisions…………………03
Not applicable/No decision made………………98 → Next activity

- **G2.07:** How much input did you have in decisions about how to use income generated from [ACTIVITY]?
  - Enter the appropriate response code from the codes listed at the bottom of the module (write one):
    Little to no input in decisions ……….............01
    Input into some decisions………..……………..02
    Input into most or all decisions…………………03
    Not applicable/No decision made……………..98 → Next activity

Regarding **G2.03**, **G2.06** and **G2.07**, please note the following:

- Code 98 ("not applicable") should be entered in the case that the decision is not made, for example crops may have been lost so no income was generated or livestock/livestock products were not sold so income was not generated. In no other case should this category be left unfilled. If the answer given for any of the questions G2.02, G2.06 or G2.07 is "not applicable or no decision made", skip to the next activity.

For rows G and H, questions **G2.07** should not be asked, skip to the next activity.
For rows I to K, questions **G2.07** and **G2.08** should not be asked, skip to the next activity.

Items in Table G2.01 – G2.07

In some cases respondents will need more explanation about what certain categories contain. In this case the enumerator can use simple examples to explain. Examples can be tailored to the specific activities undertaken by households in the survey area. Here are some examples which can be used:

- **A. Staple grain farming and processing of the harvest:** grains that are grown primarily for food consumption: For example, did you have input into decisions about what type of grains, like rice, maize, or wheat, to plant this year or in which plots they would be planted, or which seeds, fertilizer (other inputs) your family would buy?
- **B. Horticulture (gardens) or high value crop farming and processing of the harvest**: For example, did you have input into decisions about what type of cash crops or vegetables to plant this year or in which plots they would be planted, or which seeds, fertilizer (other inputs) your family would buy?
- **C. Large livestock raising and processing of milk and/or meat:** For example, did you have input into decisions about the purchase, care, or sale of large livestock such as cattle or buffaloes?
- **D. Small livestock raising and processing of milk and/or meat:** For example, did you have input into decisions about the purchase, care, or sale of small livestock such as sheep, goats, or pigs?
- **E. Poultry and other small animals raising**: For example, did you have input into decisions about the purchase, care, or sale of chickens, ducks, turkeys, quails and processing of eggs and/or meat from these livestock?
- **F. Fishing or fishpond culture**: For example, did you have input into decisions about when to fish, how to stock a fish pond, or inputs for fish culture?
- **G. Non-farm economic activities:** Small business, self-employment, buy-and-sell: For example, did you have input into purchases made for a small business or goods sold?
- **H. Wage and salary employment**: in-kind or monetary work both agriculture and other wage work: For example, did you have input into decisions about whether you or other household members will work outside the home?
- **I. Large, occasional household purchases:** For example, did you have input into purchases of expensive appliances for the house like a refrigerator or furniture? Or more valuable assets such as land or a bicycle?
- **J. Routine household purchases:** For example, did you have input into lesser household expenditures, such as those for daily needs, like food consumption? The focus of these purchase

categories are the size (small in value) and frequency of purchases (routine, day-to-day purchases), unlike the household purchase categories collected in the nutrition and health module which focuses on items that have special relevance for health and nutrition outcomes.

- **K. Obtaining agricultural inputs:** For example, did you have input in obtaining agricultural inputs such as fertilizers, feed, and services such as veterinary, advisory?

VI. MODULE G2: ROLE IN HOUSEHOLD DECISION-MAKING AROUND PRODUCTION AND INCOME

Table G2.09 – G2.20

The purpose of this module is to get an idea about men's and women's relative roles in decision-making around **Livestock activities**. Do not attempt to ensure that responses are the same between the male and female respondent. It is okay for them to be different.

Please introduce the section by telling the respondent that you would like to talk about decision making around livestock activities in their household, in order to give us insight into how the livestock assets are used by household members.

Definition of items in Table G2.08 – G2.20

LARGE RUMINANT (DAIRY LOCAL) …1 – this refers to dairy cattle that are of local/indigenous in the area. **Note**: Dairy cattle (also called dairy cows) are cows kept solely for milk production. **Note**: Select this option only when the entire herd of local cattle is kept for milk production only

LARGE RUMINANT (DAIRY IMPROVED BREEDS) …2 – this refers to dairy cattle that are either exotic or crossbreeds from exotic cattle. Dairy cattle (also called dairy cows) are cows kept solely for milk production. **Note**: Select this option only when the entire herd of improved cattle is kept for milk production only

LARGE RUMINANT (BEEF OR MIXED LOCAL) …3 - this refers to local/indigenous cattle kept for both milk and beef production (dual purpose). These are what is mostly found in smallholder farm households who keep cattle. **Note**: Select this option when the entire herd is composed of local breeds and has both cattle for milk production and beef production

LARGE RUMINANT (BEEF OR MIXED IMPROVED BREEDS)…4 - this refers to either exotic or crossbreeds from exotic cattle kept for both milk and beef production (dual purpose). These are what is mostly found in smallholder farm households who keep cattle. **Note**: Select this option when the entire herd is composed of improved breeds and has both cattle for milk production and beef production

SMALL RUMINANT (SHEEP, GOAT LOCAL)….5 - this refers to local/indigenous sheep and goats

SMALL RUMINANT (SHEEP, GOAT IMPROVED BREEDS)…6 - this refers to either exotic or crossbreeds from exotic sheep and goats

POULTRY (LOCAL)….7 - this refers to local/indigenous breeds of any type of domestic fowl, such as chicken, turkeys, ducks, geese, quails etc

POULTRY (IMPROVED BREEDS)….8 - this refers to exotic or crossbreeds from exotic breeds of any type of domestic fowl, such as chicken, turkeys, ducks, geese, quails etc

PIGS (LOCAL)…9 - this refers to local/indigenous breeds of pigs/swine

PIGS (IMPROVED BREEDS)…..10 - this refers to exotic or crossbreeds from exotic breeds of pigs/swine

CAMELS …… 11

Ask the respondent the following questions:

G2.08. Which of the following species of livestock are raised in your household – Select all the species that the respondent says the household keeps. **Note:**

- For large ruminants, if a household keeps both dairy animals and beef animals, only select **LARGE RUMINANT (BEEF OR MIXED…)** option and **not both** **LARGE RUMINANT (Dairy and LARGE RUMINANT (BEEF OR MIXED).**
- If any livestock specie that does not fall in any of the listed categories is mentioned by the respondent, select **"Other specify"** option and proceed to write the name of the said livestock specie.

G2.09: Please select the one most important species and breed type for your household livelihood – select only one livestock specie from the list, the one the respondent identifies as most important for the household livelihood

G2.09a: Reason why it is most important to your household's wellbeing/livelihood: - This is a free text field where you should type in the reason cited by the respondent as to why the selected livestock specie is important to the household.

G2.10: Please select the most important species and breed type for your own livelihood - select only one livestock specie from the list, the one the respondent identifies as most important for **his or her own** livelihood.

G2.10a: Reason why it is most important to your own livelihood: - This is a free text field where you should type in the reason cited by the respondent as to why the selected livestock specie is important for **his or her own** livelihood.

G2.11: Is there any other species and breed type that you feel is important for your livelihood that your household does NOT keep? – Select all the species that the respondent says the is important for **his or her own** livelihood but the household does not keep. **Note:**

- If any livestock specie that does not fall in any of the listed categories is mentioned by the respondent, select **"Other specify"** option and proceed to write the name of the said livestock specie. **Note:** Multiple answers accepted

G2.11a: REASON WHY YOUR HOUSEHOLD IS NOT KEEPING THIS LIVESTOCK SPECIES? -- This is a free text field where you should write the reason cited by the respondent as to why the selected livestock specie is not kept by the household.

List of activities in Table G2.12 – G2.20

A. **Animal feeding** – this entails collecting, purchasing, preparing and/ or bringing feed to animals
B. **Animal watering** - this entails collecting or bring water to animal
C. **Animal grazing** - this entails taking animal out of the farm for grazing
D. **Check animal health** - this entails observing the animal for any signs of health issues
E. **Carry out disease preventive measures** - this includes spraying, deworming, or taking animals to dip
F. **Carry out curative measures** - this includes giving medicines to heal sick animals
G. **Milking animals** - this entails drawing milk from (a cow or other animal), either by hand or mechanically

H. **Selling milk/ eggs** – this entails talking milk to a buyer or delivering it to a collection centre for the purpose of exchanging it for money

I. **Cleaning animals, shelter or utensils** – this entails removing the dirt from animal shelters and ensuring that the shelters and utensils used for handling animals are clean.

J. **Slaughter animals** - this entails killing (animals) for food

K. **Prepare animal meat, eggs, milk into food** - this entails cooking/cleaning and serving livestock product as food

L. **Breeding animals in own flock** - this entails:

1. choosing female and male animals to parent the next generation;
2. nurturing the selected parents through better care;
3. separating males and females and arrange their mating at appropriate times

Note: any or all the 3 above, should be considered as breeding animals

M. **Arrange for artificial insemination** – this entails:

1. contacting the AI provider
2. choosing the animals to parent the next generation
3. arranging to receive AI service

Note: any or all the 3 above, should be considered as arranging for artificial insemination

N. **Receiving sire service** – This entails:

1. looking for others to provide male animal for breeding
2. choosing the animals to parent the next generation
3. arrange to receive sire service

Note: any or all the 3 above, should be considered as receiving sire service

O. **Deciding how much product** from [ANIMAL] to put aside for household consumption - how much to consume versus how much to sell

P. **Marketing of live animals and products from live animals**– this entails preparing and delivering livestock (live animals) and livestock products (e.g. meat from slaughter, hides and skins etc, except milk and eggs) to a buyer or place of sales for the purpose of exchanging it for money

Q. **Selecting which species and which breeds to rear**

R. **Sharing livestock workload among household members** – this entails allocating livestock-related chores to different household members

S. **Providing this livestock as collateral to access credit**

T. **Using dung from livestock or using livestock as draft power** – this entails using dung to produce manure and applying it on the farm, it also entails using livestock for ploughing and transporting goods.

Additional activities, applicable by vaccine projects in Kenya and Uganda

U. **Vaccinating GOATS against RVF** (Rwanda) – RVF is an acronym for Rift Valle Fever. RVF) is an acute, fever-causing viral disease most commonly observed in domesticated animals (such as cattle, buffalo, sheep, goats, and camels)

V. **Vaccinating GOATS against CCPP** (Kenya) – CCPP is an acronym for Contagious Caprine Pleuropneumonia. CCP is a highly fatal disease that occurs in goats, whose clinical sines include Weakness, anorexia, cough, hyperpnea, and nasal discharge accompanied by fever.

W. **Vaccinating GOATS against PPR** (Uganda) – PPR is an acronym for Peste des petits ruminants, also known as 'goat plague'. It is a viral disease of goats and sheep characterized by fever, sores in the mouth, diarrhea, pneumonia, and sometimes death.

X. **Vaccinating CHICKEN against NCD** – NCD is an acronym for Newcastle disease. NCD is a highly contagious viral infection that affects many species of domestic and wild birds to varying degrees.

G2.12: For each of the listed livestock activities, ask the respondent whether he/she participated in the activity in the past 12 months (that is during the last [one/two] cropping seasons), from [PRESENT MONTH] last year to [PRESENT MONTH] this year

G2.13a: For each of the listed livestock activities, ask respondent how regularly they participate on the activity (pre-defined intervals e.g. Daily, two times a week etc.)

G2.13b: For each of the listed livestock activities, ask the respondent how many hours he/she spends on the activity (Based on the frequency interval selected in G2.13a)

G2.14: When decisions are made regarding [ACTIVITY], For each of the listed livestock activities, ask the respondent who is it that normally makes decisions are made regarding the activity. Note: Please fill up to 3 members. IF response is SELF ONLY (i.e. the respondent only), skip to **G2.16**

G2.15: For each of the listed livestock activities, ask the respondent how much input he/she had in making decisions about the activity

G2.16: For each of the listed livestock activities, ask the respondent to what extent he/she feel he/she can participate in decisions regarding the activity if he/she want(ed) to

G2.17: For each of the listed livestock activities, ask the respondent who would he/she prefer to make the decisions about the activity. **Note:** List up to 3 members

G2.18: For each of the listed livestock activities, ask the respondent to what extent is he/she able to access information that he/she feel is important for making informed decisions regarding the activity

G2.19: For each of the listed livestock activities, ask the respondent how much input he/she had in decisions about how much of the outputs of the activity to keep for consumption at home rather than selling

G2.20: For each of the listed livestock activities, ask the respondent how much input he/she had in decisions about how to use income generated from the activity

VII. MODULE G3(A): ACCESS TO PRODUCTIVE CAPITAL

Please introduce the section by telling the respondent that you would like to talk about assets in their household and how decisions about the assets are made, in order to give us insight into how the assets are used to empower household members.

Questions G3.01 to G3.06

G3.01.: Ask the respondent if anyone in the household currently own or cultivate land. If the answer is yes, to currently owning land or yes to cultivating land, indicate **YES** in the Tablet. If **NO**, move to ***G3.06, ITEM A***

G3.01(a): Ask the respondent how much land the household owns. Convert the total units to Acres and give the amount in acres.

G3.01(b): Enumerate in total, how much land area owned is under crops (cultivated), excluding land that is left fallow. Convert the measurements to Acres

G3.01(c): Enumerate in total, how much land area rented in is under crops (cultivated), excluding land that is left fallow. Convert the measurements to Acres

G3.02.: For cultivated land, ask the respondent who generally makes decisions about what to plant on the land, and what to do with the output from the land. **Note** that this is a 2-in-1 question so ensure that the response you pick includes both questions i.e. what to plant and what to do with the output. Select up to 3 members that generally make decision any or both decisions.

G3.03. Ask the respondent whether he/she (individual), cultivates any land solely or jointly with other household/ non-household members

G3.04. If the response to **G3.03** is any of the **Yes'**, probe further to know who generally makes decisions about what to plant on the land that the respondent him/herself cultivates, and what to do with the output from the land. **Note** that this is a 2-in-1 question so ensure that the response you pick includes both questions i.e. what to plant and what to do with the output. Select up to 3 members that generally make decision any or both decisions.

G3.05. Ask the respondent whether he/she (individually), owns any of the land owned or cultivated by their household. **Note** that this is a 2-in-1 question so ensure that the response you pick includes both questions i.e. land owned or land cultivated by their household

Table G3.06 - G3.11

Introduce the section by telling the respondent that you would like to talk about a number of items that could be used to generate income in the household. Pick one ***item*** *at a time and ask the respondent questions G3.06 to G3.11*

Definition of items in Table G3.06 - G3.11

Large ruminant (dairy) – this refers to dairy cattle. Dairy cattle (also called dairy cows) are cattle cows bred for the ability to produce large quantities of milk. Dairy cows are generally are cows kept solely for milk production.

Large ruminant (beef or mixed) - this refers to cattle kept for both milk and beef production (dual purpose). These are what is mostly found in smallholder farm households who keep cattle

Small ruminant (Sheep, goats) – this refers to sheep and goats

Poultry – this refers to all types of domestic fowl, such as chicken, turkeys, ducks, geese, quails etc

Pigs – this simply refers to pigs (swine)

Fish pond or fishing equipment – a fish pond, or fishpond, is a controlled pond (a small body of still water formed naturally or by artificial means), artificial lake, or reservoir that is stocked with fish and is used in aquaculture for fish farming.
Fishing equipment are used for fishing

Non-mechanized farm equipment (hand tools, animal-drawn plough) – these include Hand/ ox/ donkey cart; Shovel; Axe; Bush knife (panga); Hand hoe; Ox-Plough, ox-Harrow, Wheel barrow, hand Spray pump, manual Chaff cutter, manual Pulveriser machine, Milking cans etc

Mechanized farm equipment – these include tractor-ploughs, tractor-harrows, power tiller and treadle pumps, water pumps
Non-farm business equipment – these include solar panels, sewing machine, brewing equipment, fryers
House or building - these include household living quarters and other buildings owned by the household
Large consumer durables - these include refrigerators, television, sofa, gas/electric cooker, water tank
Small consumer durables - these include radio, cookware
Cell phone
Other land not used for agricultural purposes - these include pieces/plots, residential or commercial land
Means of transportation - these include bicycles, motorcycles, rickshaw, cars

G3.06 Ask the respondent whether anyone in the household currently has any **[item]**
G3.07 Ask the respondent whether he/she (individually), owns any **[item]**
G3.07a: Total number owned: Ask the respondent the total number of the **[item]** owned by the household (all in total)
G3.07b: Number owned solely: Ask the respondent the total number of the **[item]** owned by the respondent her/himself (individually)
G3.07c: Number owned jointly: Ask the respondent the total number of the **[item]** owned jointly by the respondent her/himself and any other person
G3.08 For assets owned solely by the respondent, ask the respondent what she/he can do with the asset on his/her own, without consultation. Note: select all the possible options.
G3.09 For assets owned jointly, ask the respondent who she/he co-owns it with
G3.10 For assets owned jointly with someone else, ask the respondent what she/he can do with the asset on his/her own, without consultation. **Note**: select all the possible options.
G3.11 For all assets owned by the respondent (solely or jointly), ask the respondent what his/her spouse can do with the asset on their own, without consulting the respondent. **Note**: select all the possible options.
For Non-livestock items, only question **G3.07** is asked

VIII. MODULE G3 (B): Access to credit

This module contains questions on access to credit. A few things to consider:

- Lending sources can be adapted to the country context, however it is important that they are distinguished from each other and there is no double counting. If local language does not distinguish between categories, they may be combined into one. For instance, if a non-governmental organization and group-based microfinance or lending is the same in the local context (if all group-based microfinance is run through NGOs), then it is okay to combine the categories into one. In this case there would be five categories instead of six to ask the respondent. Never should the same lending source be counted under multiple categories. If you feel that what the respondent describes could belong under two or more categories, use judgment and put it under the one category you feel is best.
- The recall timeframe is within the last year; however, if a credit source has been ongoing for more than 1 year (i.e. a multi-year loan that began 2 years ago) it should be counted.
- "In-kind" refers to credit given in the form or goods, commodities or services as opposed to cash.
- "Informal lenders" refers to those like moneylenders or others that are NOT included in one of the other categories of credit.

- Note that question **G3.08** is not included in the calculation of the pro-WEAI. However, we do recommend that you ask it, as it allows you to determine whether individuals are able to borrow from a particular source, even if they did not try to in the last year. This is important for programming, because it indicates whether there is a credit constraint in terms of unmet demand for credit.
- In the case of **G3.09**, in some cases the individual may not know if other members of the household have accessed or used a specific type of credit, and in this case the response 97 "Don't know" can be entered.
- For **G3.10**, **G3.11** and **G3.12** enter up to 3 member IDs; make sure to probe the respondent and ask "is there anybody else?"
- Question **G3.13** includes digital financial services (Mobile money).

IX. MODULE G4: Time allocation

The purpose of this module is to get an idea about how men's and women's time is spent. Types of activities and their duration can be used in economic as well as in social analysis, e.g. women's contribution in economic activities; the value of home production and the informal sector; productivity; time poverty and others. We are particularly interested in agricultural activities such as farming, gardening, and livestock raising whether in the field or in the homestead. We are also interested in how much time is spent caring for children, especially if it happens while the respondent is doing other activities.

G4.01: Please record a log of the activities for the individual in the last complete 24 hours (starting yesterday morning at 4 am, finishing 3:59 am of the current day).

G4.02: Mark this box if the respondent also cared for children while doing another activity. For this question, caring for children includes both "active" caring (example: feeding, bathing child) and "passive" caring (example: collecting water while carrying a child). Note: This should be asked as the primary activities are being filled out.

- The time grid begins at 4 am to account for respondents that wake up very early. It is most helpful to fill this table while engaging in a conversation with the respondent, rather than asking them what they were doing at 4:15, 4:30, 4:45 and so on. First, it is helpful to establish what time the respondent woke up and went to bed, so then the waking hours are what remains.
    - The enumerator should first ask the respondent what time he/she woke up the previous day, and then fill in any time before that as "sleeping and resting".
    - Next, the enumerator can ask what time he/she went to sleep the previous day, and then fill in any time after that as "sleeping and resting". Doing these first two steps in the beginning sets the time boundaries for the day that need to be accounted for.
- After establishing the wake-up and sleeping times, the enumerator can then ask the respondent what they did next, and for how long, and so on until the entire day is filled out. For example, ask after you got up what did you do? The respondent then narrates what he or she did and for how long. The enumerator's task is then to identify the correct code associated with each activity and to enter the response in the correct time interval. Next, the enumerator asks, did you also care for children while doing this activity? The enumerator should check the box if the respondent was also caring for a child while doing something else. Even if the respondent is not actively caring or interacting with the child

(e.g, the child is sleeping or doing something else), for as long as they are responsible for the child during that time, it should be counted as "Yes" and the box should be checked for the appropriate time interval.

- Depending on the context, it may also be useful to use common time markers to help respondents recall activities in relation to other events that happen predictably throughout the day (i.e. always take lunch at 1, prayer time, sunrise/sunset, etc).
- The time intervals are marked in 15 min intervals and one activity can be marked for each time period by writing the activity code in the box. Do not leave any of the time intervals blank. If an activity is done over longer periods of time (beyond 15 minutes), the same activity code should be repeated for the duration of the activity. For example, if activity "E" was done for 1 hour, then "E" should be marked in four consecutive boxes.
- Make sure that your marks are very clear. You should never have more than one activity marked for a time period. If the respondent was doing multiple things (for instance eating breakfast and listening to the radio) ask the respondent which activity was the primary focus, and enter that activity code for the appropriate time period. If the secondary activity is caring for a child, make sure to also check the box for G4.02.
- If an activity is completed for the major part of 15 minutes (up to 8 minutes), then mark the entire box for that activity. However, if an activity is only completed for the minor or lesser part of 15 minutes (less than 8 minutes) then simply do not count the activity in the table. For example, if the respondent ate/drank for 20 minutes, only one box should be filled (15 minutes is accounted for and the remaining 5 minutes is lost because it does not reach 8 minutes). If however, the respondent ate/drank for 25 minutes, then two boxes should be filled (15 minutes in the first box and 10 minutes in the second box). A box can never be split.
- When a respondent describes many activities in a short period, such as their morning routine, use your best judgement to figure out which categories the majorities of activities belong in and fill that time grid for the period.
- Never leave a 15 minute increment blank. All 24 hours should be accounted for.

Example: In the diagram below, we can see that the respondent was sleeping or resting up till 5:30 am. At 5:30 am he/she listened to the radio for 30 min, followed by eating breakfast for 15 min and bathing and dressing for 15 min. At 6:30 am he/she started working in a shop, which he/she continued for the next one and a half hours.

		**Night**								**Morning**				**Day**															
		4:00				5:00				6:00				7:00				8:00				9:00				10:00			
**G4.01** Activity **(WRITE ACTIVITY CODE)**		A	A	A	A	A	A	C	C	P	P	B	B	H	H	H	H	H	A	A	K	K	K	J	J	J	J	J	J
**G4.02** Did you also care for children?	YES ...... CHECK BOX<br>NO.... LEAVE BLANK	☐	☐	☐	☐	☐	☐	✔	✔	✔	✔	✔	✔	☐	☐	☐	☐	✔	✔	☐	☐	☐	☐	✔	✔	✔	✔	✔	✔

Determining Activities ✔

The list of activities is quite general so it may be the case that something the respondent has done is not described among the activities. First think if the activity fits in any other of the pre-printed activities. If it does not, then use

the category Other, specify. Try to describe it in as detailed a manner as possible. If there is more than one activity that fits into the “Other” category, make sure there is a separate description for each activity.

Formal **work** and **school** override other activities. Personal care, eating, reading, travelling, etc. during working and school hours is always marked as work/school. However, an official break is recorded as eating, personal care, shopping or whatever has been done. Many activities, like shopping, are linked with **travels**. If such travelling is the longest part of a 15 minutes period, it should be noted as travelling. Specific travel to and from work or school should be recorded as **commuting** (activity M), not travelling.

Note: Activities G-L cover include ALL aspects of the category. For example, with respect to large livestock raising (such as a cow) this means time spent feeding the cow, milking the cow, and processing and selling the milk from the cow.

Activity	Specification
A: Sleeping and resting	Includes resting, e.g. trying to sleep.
B: Eating and drinking	Includes time spent on consuming food and drink. Record eating/drinking with a social component as "social activities and hobbies. Eating just snacks with friends or when watching TV is not regarded as the main activity.
C: Personal care	May include bathing, getting dressed, brushing teeth/hair, etc. Record purchased services like haircutting as “shopping/getting service”.
D: School	Personal care and shorter breaks during school hours are treated as school.
E: Work as employed	Includes personal care, eating, traveling, reading, etc. during the working hours which are part of your income generating activities (i.e. you are sent across town to attend a meeting, or you are reading for work purposes) but excludes commuting to and from work (record under “traveling and commuting”).
F: Own business work	Includes own account work and household related businesses, except farming, fishing and textile work even for selling.
G: Staple grain farming and processing of the harvest	Grains that are grown primarily for food consumption (rice, maize, cassava, millet, wheat). This includes post-harvest processing in the field and in the homestead regardless of whether it is income generating or for home consumption.
H: Horticulture (gardens) or high value crop farming and processing of the harvest	Includes small-scale food production in the garden for own consumption and selling. This includes post-harvest processing in the field and in the homestead regardless of whether it is income generating or for home consumption.
I: Large livestock raising	Feeding, raising, grazing, taking cattle, buffaloes, etc. to market; processing products and taking milk to market
J: Small livestock raising	Feeding, raising sheep, goats, pigs and taking them or their products to market
K: Poultry and other small animals	Feeding, raising, chickens, ducks, turkeys, guinea fowl, and taking them or their products to market
L: Fishpond culture	Includes fishing for own consumption and selling, but excludes fishing just for fun (record as “social activities and hobbies”).
M: Commuting	Travels to and from work or school)
N: Shopping/getting service (including health services	Includes paid personal care, like haircutting, visit to the doctor or health facility (obtaining health services), car servicing and banking, etc. Any traveling linked to shopping will be noted as travels.
O: Weaving, sewing, textile care	Includes textile work for selling and own consumption, but excludes repairing of textiles (note as “domestic work”).

Activity	Specification
P: Cooking	Includes time spent getting food at market (but not transport time, which is counted under transport), preparing food to cook, time cooking, and time cleaning up after. Does not include time spent harvesting crops (include in "farming/livestock/fishing").
Q: Domestic work	Includes all unpaid domestic work such as fetching water and firewood, cleaning, washing clothes and other household chores (excluding cooking). Paid domestic work is counted as "work as employed."
R: Caring for children	Caring for children involves both "active" and "passive" caring.
S: Caring for adults (sick, elderly)	Includes unpaid care for all persons at home as well as outside home. Paid care is counted as "work as employed."
T: Traveling (not for work or school)	Travel includes all travels, except commuting and travels on working hours. Includes walking if the purpose is not exercising. Longer journeys will be separated by activities like eating, personal care, etc.
U: Exercising	All kind of physical sport activities including walking, if the purpose is not moving from one place to another (which is counted as "traveling and commuting")
V: Social activities and hobbies	This category captures any social activities, such as sitting with family, visiting friends, talking on the phone with friends, visiting a drinking spot or restaurant with friends, going to watch sporting activities etc. This category also encompasses conjugal activities if they are not for paid work (otherwise can be captured as "work as employed" or "own business"). Also includes gardening, fishing and other production activities if they are just for fun. All kind of physical sport activities including walking, if the purpose is not moving from one place to another (which is counted as "traveling and commuting").
W: Religious activities	Include attending services, praying or other religious activities/ceremonies. Note that if the individual is a Pastor, Imam or other person that does this as their occupation/work, it should be counted in the "work as employed" category and not as a religious activity.
Z: Watching TV/listening to radio/reading	Includes watching TV, listening to a radio and/ or reading
Other (specify)	

- **G4.03:** In the last 24 hours did you work less than usual, about the same as usual or more than usual? For this question, we want to know how the respondent's previous day compared to their usual routine.
    - For this question, work is defined as all livelihood activities done at home or outside the home, paid or unpaid, including domestic tasks and chores. We are only interested in the total amount of time spent working (the respondent's perceived "workload"), so it is fine if the respondent says response "about the same as usual, but on different tasks".

The last part of this section is only applicable to female respondents with young children. If the respondent is male, the enumerator can move to the next module.

- **Note: FOR FEMALES ONLY: DOES RESPONDENT HAVE A CHILD UNDER 5 YEARS OLD? : This is for the enumerator to fill out (check roster if necessary). DO NOT ASK RESPONDENT**
    - If the respondent is female, the enumerator should check the roster to verify whether the respondent has a child under 5 years old. Since this information was already collected in the roster, we do not advise asking the respondent this question to minimize respondent fatigue.
- **G4.04a**. Ask the responded whether they can take a child with her if sheu wanted to do something livelihood-related, training-related or self-care
- **G4.04b:** If you wanted to do something (livelihood-related, training-related, self-care) and could not take your child with you, is there someone who could care for your child in your absence?

- For these last two questions, we are interested in the respondent's access to childcare. Participation in many types of development projects require spending additional time for training, group meetings, etc, and so access to alternative childcare providers can determine how easily women can participate in such projects.

- **G4.05**: Enter up to three member IDs of the people who can care for the respondent's child/children in her absence. If the person is not a member of the household, please select other non-household members as appropriate.

X. MODULE G5: Group membership

The purpose of this module is to get information regarding men's and women's access to social capital.

A few things to note:

- Note that groups in the community can be either formal or informal and customary groups.
- Note that being an "**active**" member of a group should be defined by the respondent (i.e. his/her subjective idea of what being a member constitutes). If asked by the respondent, you may indicate that "active" membership could constitute attending meetings, paying a user fee, holding a leadership position within the group, etc. However, explain that there is too much variation in group type to have a standard definition for an active member so encourage the respondent to use his/her own judgment.
- Note that "community" is left to the respondent to define and may be groups within his/her own village or encompass a larger geographic range including a nearby village or city.
- Note that many groups have multiple activities. For instance, an agricultural group may have a microfinance component. When this is the case, choose the group category that represents the primary activity. If the agricultural group provides many extension services, including microfinance, then agricultural group, not credit or microfinance group, should be selected. Ask the respondent to describe the group in greater detail if you are unclear.
- A "religious group" may include going to church, the mosque, etc. or being a member of a small prayer or religious discussion group.
- If a certain group is not contextually appropriate, it' may be replaced with the appropriate group in the same category; if no replacement exists it may be omitted from the questionnaire. Whether or not to omit a group should be decided during the training; a group should never be omitted when conducting a questionnaire.

XI. MODULE G6: Physical mobility

The purpose of this module is to get information regarding the places men and women go outside the home. **The questions G6.01-G6.07 are required**.

- **Q6.01:** This question refers to an urban center or the municipality of a town. Please use the official census or government definition of an urban center or municipality that is used in your specific setting. The questions G6.02-G6.07 should ONLY BE ASKED IF RESPONDENT IS FEMALE.
- This part of the module is optional (i.e. **G6.02**, **G6.05**, **G6.06** and **G6.07**)
- Note: Place I "Outside your community or village" is a broad category of places (may include, but not limited to an urban center).
- Note: If the female respondent says that ***she does not want to go*** a [PLACE], then the data collector should ask, "***If you wanted to go,*** who would usually decide whether you can go to [PLACE]?"

XII. MODULE G7: Intrahousehold relationships

The purpose of this module is to get information regarding mutual respect and conflict between husband and wife or between the two household decisionmakers.

- **Note: Rows A and B are REQUIRED**; rows C and D are OPTIONAL. Add rows to ask about people outside the household like a mother in law that lives outside household and co-wives in polygamous households.
- **G7.01**: IMPORTANT: enter household member ID # that corresponds the person listed the row
- **G7.06:** DO NOT READ ALOUD TO RESPONDENT. The enumerator should check the roster/cover page to see if the person that the respondent is referring to is also the other respondent. The respondent may not necessarily know this information.
- **G7.07**: it is HIGHLY RECOMMENDED to ask this for FEMALE RESPONDENTS in contexts with polygamy

XIII. MODULE G8(A): Autonomy in decision making

The purpose of the module is to understand the motivations behind men's and women's actions in different activities.

- Each situation (e.g., A1, A2, A3, A4) describes a different type of motivation. Motivation #1 is doing something because you don't have a choice. Motivation #2 is doing something because you will get in trouble with someone if you act differently. Motivation #3 is doing something so others don't think poorly of you. Motivation #4 is doing something because you personally think it is the right thing to do.
- Enumerators should read aloud each story, the subsequent questions, and the response codes. Make sure to change the names to reflect commonly used male and female local names. The names should be male/female depending on the sex of the respondent, so that the male names are in the stories read to the male respondent, and female names are in the stories read to the female respondent. The order of topics a-d should be randomized, and within each topic, the order of stories 1-4 should be randomized.
- We recommend asking all questions in this section (G8.01, G8.02, G8.03) to capture a more precise indicator on autonomy. However, some users may choose to drop G8.02 and G8.03 if there is a large risk of nonresponse, or if this is not a priority indicator for the project. In this case, only G8,01 will be collected "Are you like this person?". Note that dropping the follow-up questions (G8.02 and G8.03) is likely to identify more people as disempowered.

XIV. MODULE G8(B): NEW General self-efficacy scale (OPTIONAL)

The purpose of this module is to get information regarding a respondent's belief in themselves (self-confidence) and their ability to achieve the goals they set for themselves.

- Think about how each statement relates to your life, and then tell me how much you agree or disagree with the statement on a scale of 1 to 5, where 1 means you "strongly disagree" and 5 means you "strongly agree."
- **Note: It may be useful to randomize the order of statements**

XV. MODULE G8(C): Life satisfaction (OPTIONAL)

- The following questions ask how satisfied you feel with your life as a whole, on a scale from 1 to 5, where 1 means you feel "very dissatisfied" and 5 means you feel "very satisfied."

MODULE G9: Attitudes about domestic violence

The purpose of this module is to get information on the attitudes and beliefs regarding tolerance for the use of violence against women in intimate relationships. The questions in this module do not ask about personal experience but rather are getting at attitudes about domestic violence. It is important that this module be at the end of the survey for sensitivity reasons.

- IMPORTANT: These questions are required for both men and women
- If you are asking a respondent about a non-husband, ask question in same way
-

XVI. End of the interview

At the end of the interview:

- Sincerely thank the respondent for his/ her time and
- Fill up the question on the outcome of the interview (G1.05)
- Then capture the household's GPS coordinate
- Remember to keep the interview data safe i.e. save and upload the data if the interview was conducted using CAPI, or keep the paper copy of the questionnaire safe and hand it to the supervisor for filling

7.
