# Supplementary material for: Women’s empowerment and intra-household gender dynamics and practices around sheep and goat production in South East Kenya
Source: PLoS One. 2022 Aug 4;17(8):e0269243. doi: 10.1371/journal.pone.0269243 (PMC9352016; doi:10.1371/journal.pone.0269243)
Supplement: S2 Appendix — (PDF) [file pone.0269243.s002.pdf]

ILRI
INTERNATIONAL LIVESTOCK RESEARCH INSTITUTE

The Women's Empowerment in Livestock Index:

Vital progress towards gender equality

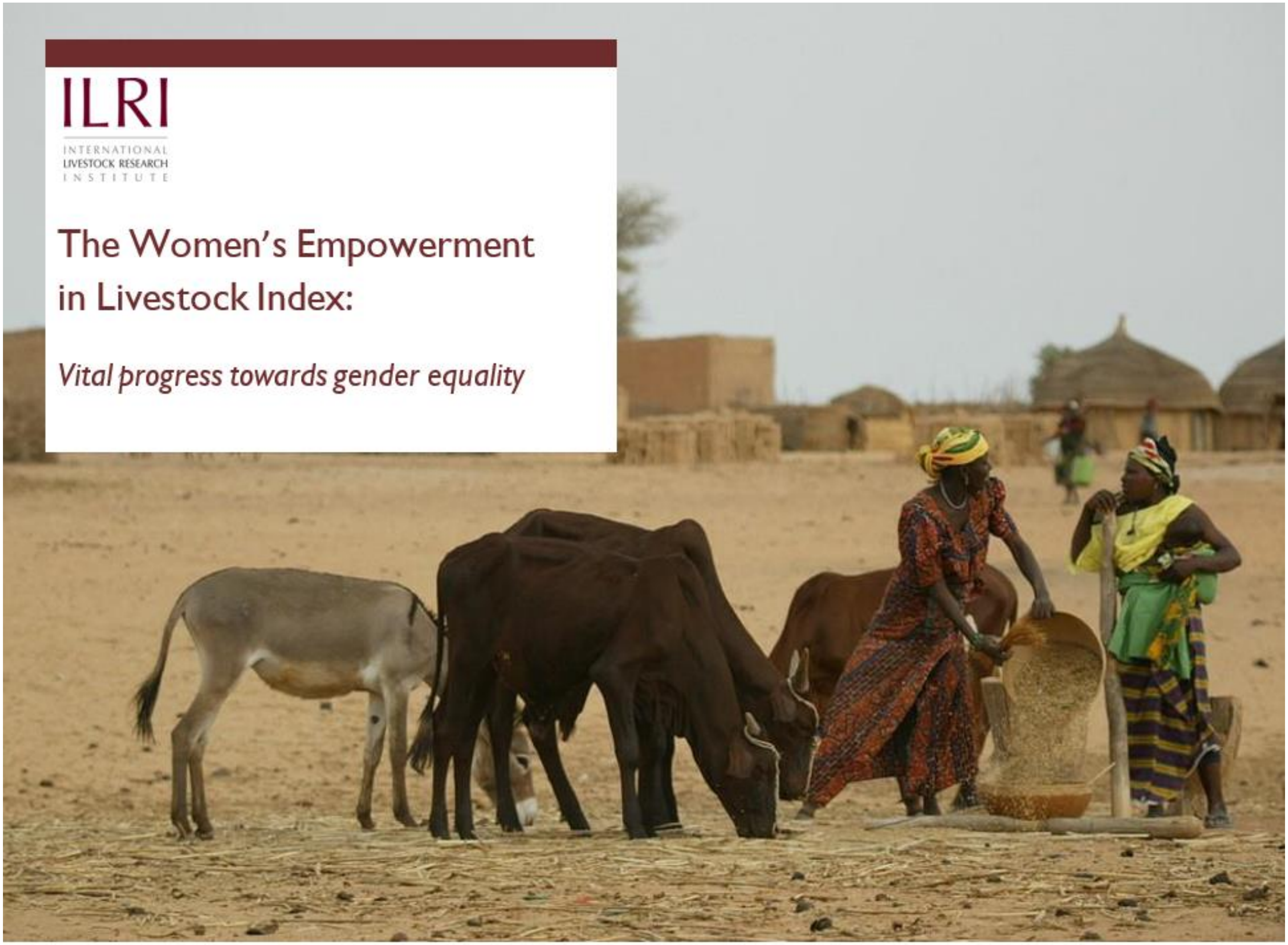

Contact person: Alessandra Galiè, PhD | Senior Gender Scientist | International Livestock Research Institute | ilri.org | Email: a.galie@cgiar.org

THE WOMEN'S EMPOWERMENT IN LIVESTOCK INDEX (WELI)

An introduction: The WELI is a standardized measure to capture the empowerment of women involved in the livestock sector. Diverse strategies exist to empower women, yet these strategies are difficult to prioritize without a reliable and adapted means to measure women's empowerment. One quantitative measure is the Women's Empowerment in Agriculture Index (WEAI). Despite its reliability in certain agricultural contexts, the WEAI requires adaptation in settings where livestock farming is the dominant form of livelihood. Using the WEAI as a starting point, a multidisciplinary team of researchers from the International Livestock Research Institute (ILRI) and Emory University developed the Women's Empowerment in Livestock Index (WELI), a new index to assess the empowerment of women in the livestock sector.

Contacts:

Alessandra Galiè, PhD, Senior Gender Scientist, International Livestock Research Institute, Email: a.galie@cgiar.org

Nils Teufel, PhD, Senior Agricultural Economist, International Livestock Research Institute, Email: n.teufel@cgiar.org

DRAFT

QUALITATIVE COMPONENT

Complementary research: Taking into account the complexity and contextual aspects of empowerment we recommend that 2 rounds of qualitative research are undertaken to complement the WELI survey and provide depth of information.

The first round of qualitative research pre-survey:

Timing: This first round needs to be completed before the survey starts.

Aims: 1. provide an understanding of how empowerment is understood locally; 2. Help phrase the WELI in a contextually appropriate manner (e.g. using local examples); 3. Prepare the respondents for the topics to be covered in the survey; 4. Validate the WELI, especially for the new indicators (see 'analysis' below)

Tools: It consists of focus group discussions on 'local understandings of empowerment'.

Methodology: 1 FGD of 8 to 12 adult women – 1 FGD of 8 to 12 adult men. If there is considerable ethnic, livelihood, income etc. heterogeneity (i.e. differences in other social markers) within the sample site that is likely to prevent some individuals from freely expressing their opinion, then additional focus groups should be done organizing the group based on ethnic/income/livelihood group. In cases when it does not prevent individuals from freely expressing their opinion, such heterogeneity of social markers can be leveraged to spark interesting conversations and therefore participants can be involved in 1 FGD only (note will need to report these social markers because they may help explain some of the responses – see below).

Questions:

a. What does the word 'empowerment' mean to you?

   Note: the word 'empowerment' needs to be translated in the local language with a 'neutral word' i.e. a word that possibly avoids conveying associations to 'wealth, 'education' etc. The definition you can keep in mind when looking for a local word is:

DRAFT

‘empowerment is the process by which an individual acquires the capacity of living the life that she or he values’ i.e. similar to ‘ability to determine own life’.

In case there is no knowledge of the word ‘empowerment’, then the following ‘exercise’ can be done: Ask: ‘Imagine what your **ideal** life (or a life you would like for yourself) would look like 10 years from now…. (silent exercise; each one thinks for themselves). After a couple of minutes ask: ‘Now imagine **realistically** how your life is mostly likely going to be like 10 years from now’. After some minutes ask: ‘what needs to happen to you in order to make your ideal/dream life come true?’ discuss this last question as a group and write down comments e.g. ‘I need to be…, I need to get...’; ‘My community needs to allow me to....’. Use this idea of ‘what needs to happen to you to realize your ideal life’ as a concept of empowerment to discuss the following questions.

b. What are the characteristics of an empowered man?

Note: To keep people engaged you can draw a neutral body on a flipchart and add the mentioned characteristics

c. What are the characteristics of an empowered woman?

Note: To keep people engaged you can draw a neutral body on a flipchart and add the mentioned characteristics

You can also tailor these questions to your group e.g. ‘what are the characteristics of an empowered chicken woman farmer?’….

Note taking: When taking notes, please follow good practices of gender analysis e.g. you may want to keep track of who is saying what for the analysis to be richer – while guaranteeing anonymity (e.g. you can record at the beginning of the FGD the main characteristics of the respondents: Respondent 1: older woman farmer, illiterate, from XX ethnicity; Respondent 2: young woman livestock farmer, single, educated…and then refer to R1 says: ‘in my experience…’ R2: ‘according to me….’)

Analysis: The analysis can be undertaken utilizing standard qualitative analysis tools (e.g. consensus analysis on indicators of empowerment common to all respondents and divergent opinions). One research question you can address with your findings is e.g.: ‘what are the differences between the characteristics of an empowered man and an empowered woman?’; ‘is empowerment different for younger men? And younger women’? etc

The findings could reveal some indicators of empowerment that are important locally but not included in the survey. These new indicators could be added as rows to existing tables (e.g. if an activity such as ‘managing local pasture conservation areas’ is a key indicator of empowerment, such activity could be added in a new row to the table G3.12-G3.20.). Please, be aware, however, that if you remove sections of the survey, the comparability of its results (vis-a-vis the pro-WEAI or WELI used elsewhere) is limited.

DRAFT

Later on, the findings can be used to better interpret the results that will be later generated from the survey by providing more depth to the views of the respondents.

The second round of qualitative research post-survey:

Analysis of the survey data may raise some questions that need to be answered as part of a conversation (e.g. you received an answer that is unexpected, and you want to understand the answer better; or the survey shows a trend and you want to understand the mechanisms behind that trend). You can arrange FGDs to discuss open-ended questions about the issues you identified in the survey that expand your understanding: e.g. ‘the survey shows that…can you tell me more about this...’, or ‘in the survey most people stated that XXX. In your opinion, why ...’. The findings from this qualitative analysis will help better understand the index and also provide depth to the answers.

DRAFT

Informed Consent for Women's Empowerment in Livestock Index (REQUIRED)

SAMPLE

Informed Consent: *Before beginning the interview, it is necessary to introduce the household to the survey and obtain their consent to participate. Make it clear to them that their participation in the survey is voluntary. Please read the following statement in the language of interview:*

Thank you for the opportunity to speak with you. We are a research team from [XXXX] working in collaboration with [XXXX]. We are conducting a survey to learn about livestock, food security and wellbeing of households in this area. You have been selected to participate in an interview which includes questions on topics such as your family background, decision making processes, livestock production, asset ownership and income earning activities. The survey includes both a section to be asked about the household generally, in addition to sections which will be asked to a primary adult male and female in your household if applicable. These questions in total will take approximately 1.5 hours to complete and your participation is entirely voluntary. If you agree to participate, you can choose to stop at any time or to skip any questions you do not want to answer. Your answers will be completely confidential; we will not share information that identifies you or your household with anyone (including location). After entering the questionnaire into a data base, we will destroy all information such as your name which will link these responses to you.

We will also interview other households in your community and in other parts of [XXXX]. After we collect all the information, we will use the data to make a study about how various programs can be most helpful to the people in this area. Do you have any questions about the study or what I have said? If in the future you have any questions regarding study and the interview, or concerns or complaints we welcome you to contact [XXX], by calling [XXX]. In addition, you can contact the head of Ethics Review at [XXX]. We will leave one copy of this form for you so that you will have record of this contact information and about the study.

Please ask the participants (male and female) if they consent to the participation in the study (check one box):

Participant 1:	YES	☐	NO ☐	Participant 2:	YES	☐	NO ☐

I ___________________________, the enumerator responsible for the interview taking place on _________________, 20XX certify that I have read the above statement to the participant and they have consented to the interview. I pledge to conduct this interview as indicated on instructions and inform my supervisor of any problems encountered during the interview process.

If the household does not give consent to all of the data collection, stop the interview and inform your team leader. Team leaders will discuss the reason for this refusal and decide whether a partial data collection is possible for this household.

Consent form approved by IFPRI IRB on August 16, 2011 and by UNCST on August 18, 2011.

DRAFT

Informed Consent for Women's Empowerment in Livestock Index

SAMPLE

DUPLICATE: *Enumerator: Leave a copy of this consent with the household.*

Informed Consent: *Before beginning the interview, it is necessary to introduce the household to the survey and obtain their consent to participate. Make it clear to them that their participation in the survey is voluntary. Please read the following statement in the language of interview:*

Thank you for the opportunity to speak with you. We are a research team from [XXXX] working in collaboration with [XXXX]. We are conducting a survey to learn about livestock, food security and wellbeing of households in this area. You have been selected to participate in an interview which includes questions on topics such as your family background, decision making processes, livestock production, asset ownership and income earning activities. The survey includes both a section to be asked about the household generally, in addition to sections which will be asked to a primary adult male and female in your household if applicable. These questions in total will take approximately 1.5 hours to complete and your participation is entirely voluntary. If you agree to participate, you can choose to stop at any time or to skip any questions you do not want to answer. Your answers will be completely confidential; we will not share information that identifies you with anyone. After entering the questionnaire into a data base, we will destroy all information such as your name which will link these responses to you.

We will also interview other households in your community and in other parts of [XXXX]. After we collect all the information, we will use the data to make a study about how various programs can be most helpful to the people in this area. Do you have any questions about the study or what I have said? If in the future you have any questions regarding study and the interview, or concerns or complaints we welcome you to contact [XXX], by calling [XXX]. In addition, you can contact the head of Ethics Review at [XXX]. We will leave one copy of this form for you so that you will have record of this contact information and about the study.

Please ask the participants (male and female) if they consent to the participation in the study (check one box):

Participant 1:	YES	☐	NO ☐	Participant 2:	YES	☐	NO ☐

I ___________________________, the enumerator responsible for the interview taking place on _________________, 20XX certify that I have read the above statement to the participant and they have consented to the interview. I pledge to conduct this interview as indicated on instructions and inform my supervisor of any problems encountered during the interview process.

If the household does not give consent to all of the data collection, stop the interview and inform your team leader. Team leaders will discuss the reason for this refusal and decide whether a partial data collection is possible for this household.

Consent form approved by IFPRI IRB on August 16, 2011 and by UNCST on August 18, 2011

DRAFT

DRAFT

DRAFT

LISTING OF SURVEY MODULES

HOUSEHOLD LEVEL QUESTIONNAIRE		INDIVIDUAL LEVEL QUESTIONNAIRE	
**Module A**	Household identification	**Module G1**	Individual identification
**Module B**	Household listing and demographics	**Module G2**	Role in household decision-making
		**Module G3(A)**	Access to productive capital
		**Module G3(B)**	Access to Financial services
		**Module G4**	Time allocation
		**Module G5**	Group membership
		**Module G6**	Physical mobility
		**Module G7**	Intrahousehold relationships
		**Module G8(A)**	Autonomy in decision making
		**Module G8(B)**	New general self-efficacy scale
		**Module G8(C)**	Life Satisfaction
		**Module G9**	Attitude about Domestic Violence

DEFINITION OF HOUSEHOLD AND RESPONDENTS (REQUIRED)

A **household** is a group of people who live together and take food from the "same pot." In our survey, a household member is someone who has lived in the household at least 6 months, and at least half of the week in each week in those months. Even those persons who are not blood relations (such as servants, lodgers, or agricultural laborers) are members of the household if they have stayed in the household at least 3 months of the past 6 months and take food from the "same pot." If someone stays in the same household but does not bear any costs for food or does not take food from the same pot, they are not considered household members. For example, if two brothers stay in the same house with their families but they do not share food costs and they cook separately, then they are considered two separate households. Generally, if one person stays more than 3 months out of the last 6 months outside the household, they are not considered household members. We do not include them even if other household members consider them as household members.

Exceptions to these rules should be made for:

Consider as household member:

- A newborn child less than 3 months old.
- Someone who has joined the household through marriage less than 3 months ago.
- Servants, lodgers, and agricultural laborers currently in the household and will be staying in the household for a longer period but arrived less than 3 months ago.

<u>*Do not*</u> consider as household member:

- A person who died very recently though stayed more than 3 months in last 6 months.
- Someone who has left the household through marriage less than 3 months ago.
- Servants, lodgers, and agricultural laborers who stayed more than 3 months in last 6 months but left permanently.

This definition of the household is very important. The criteria could be different from other studies you may be familiar with, but you should keep in mind that you should not include those people who do not meet these criteria. Please discuss any questions with your supervisor.

DRAFT

The **primary and secondary respondents** are those which are self-identified as the primary members responsible for the decision making, both social and economic, within the household. They are usually husband and wife, however can also be another member as long as there is one male and one female aged 18 and over. It may also be the case that there is only a primary respondent if that person is a female and there is no adult male present in the household.

DRAFT

MODULE A. HOUSEHOLD IDENTIFICATION (REQUIRED)

**Household Identification**	**Code**
**A01**. Household Identification:	
**A02**. Village:	
**A03**. Sub-county:	
**A04**. District:	
**A05**. Cell phone number:	

**Interview details**	**Code**
**A06**. Start time of interview (hh:mm)	:
**A07**. End time of interview (hh:mm)	:
**A08**. Name/code of enumerator:	
**A09**. Date of visit (dd/mm/yyyy):	/ /

DRAFT

MODULE B. HOUSEHOLD LISTING AND DEMOGRAPHICS (REQUIRED). ***Enumerator: Ask these questions about all household members.***

First, we would like to ask you about each member of your household. Please list the names of everyone considered to be a member of this household, starting with the primary respondent.

ID CODE	Name of household member?<br><br>[start with primary respondent, continue with the secondary respondent, and other members in descending order of age]	What is [NAME's] sex?<br><br>1 = Male<br>2 = Female	What is [NAME's] relationship to the primary respondent?<br><br>**CODE 1**	What is [NAME's] age?<br><br>(in complete years)
	**B01**	**B02**	**B03**	**B04**
1				
2				
3				
4				
5				
6				
7				
8				
9				
10				
11				
12				
13				
14				
15				

Code 1 (B03) Relationship to primary respondent

Primary respondent................1	Brother/sister………………. 7	Mother/father-in-law……..……………………………………….12
Spouse…………………….........2	Nephew/niece……………………8	Cousin of primary respondent's spouse ……...………………….....13
Son/daughter………..………….3	Nephew/niece of spouse……….9	Other relative…………………...14
Son/daughter-in-law…………...4	Cousin of primary respondent....10	Maid…………………………………………………………………...15
Grandson/granddaughter ……5	Brother/sister-in-law……….........11	Other relationship (specify)..16
Mother/Father...…………………..6		

DRAFT

MODULE G. WOMEN'S EMPOWERMENT IN LIVESTOCK INDEX – Pilot WELI Version

Note to survey designers: The information in module G1 can be captured in different ways; however there must be a way to: (a) identify the proper individual within the household to be asked the survey, (b) link this individual from the module to the household roster, (c) code the outcome of the interview, especially if the individual is not available, to distinguish this from missing data, and (d) record who else in the household was present during the interview. This instrument must be adapted for country context including adding relevant examples and translations into local languages when appropriate.

Note to enumerators: *This questionnaire should be administered separately to the primary and secondary respondents identified in the household roster of the household level questionnaire. You should complete this coversheet for each individual identified in the "selection section" even if the individual is not available to be interviewed for reporting purposes. For some surveys (such as those focusing on nutrition outcomes), the female respondent may be the beneficiary woman or mother or primary caregiver of the index child (also the respondent for the pro-WEAI nutrition module). Please make sure that she is also the person interviewed for this questionnaire and that the male respondent is her spouse/partner (if applicable).*

Please double-check to ensure:

- You have completed the roster section of the household questionnaire to identify the correct primary and/or secondary respondent(s);
- You have noted the household ID and individual ID correctly for the person you are about to interview;
- You have gained informed consent from the individual in the household questionnaire;
- You have sought to interview the individual in private or where other members of the household cannot overhear or contribute answers.
- Do not attempt to make responses between the primary and secondary respondents the same—it is okay for them to be different.

DRAFT

MODULE G1. INDIVIDUAL IDENTIFICATION

**G1.01. HOUSEHOLD IDENTIFICATION:**	☐☐☐☐☐☐	**G1.04 TYPE OF HOUSEHOLD**	MALE AND FEMALE ADULT .... 1<br>FEMALE ADULT ONLY 2
**G1.02. NAME OF RESPONDENT CURRENTLY BEING INTERVIEWED (ID CODE FROM ROSTER IN SECTION B HOUSEHOLD ROSTER):**	☐☐	**G1.05. OUTCOME OF INTERVIEW:**<br>**CIRCLE ONE**	COMPLETED .... 1<br>HOUSEHOLD MEMBER TOO ILL TO RESPOND/COGNITIVELY IMPAIRED .... 2<br>RESPONDENT NOT AT HOME/TEMPORARILY UNAVAILABLE .... 3<br>RESPONDENT NOT AT HOME/EXTENDED ABSENCE .... 4<br>REFUSED .... 5<br>COULD NOT LOCATE 6
**SURNAME, OTHER NAME:** ____________________			
**G1.03. SEX OF RESPONDENT:**<br><br>**G1.03a. WHO ARE YOU INTERVIEWING?**	MALE .... 1<br>FEMALE .... 2<br><br>HUSBAND ....1→ **G1.05**<br>INDEX WOMAN 2	**G1.06. ABILITY TO BE INTERVIEWED ALONE:**<br>**CIRCLE ONE**	ALONE .... 1<br>WITH ADULT FEMALES PRESENT .... 2<br>WITH ADULT MALES PRESENT .... 3<br>WITH ADULTS OF BOTH SEX PRESENT .... 4<br>WITH CHILDREN PRESENT .... 5<br>WITH ADULTS OF BOTH SEX AND CHILDREN PRESENT 6

DRAFT

HOUSEHOLD IDENTIFICATION (EACH MODULE (G2-G9) MUST BE LINKED WITH A HH AND RESPONDENT ID)

HOUSEHOLD ID						
RESPONDENT ID						

MODULE G2: ROLE IN HOUSEHOLD DECISION-MAKING AROUND PRODUCTION AND INCOME

Now I'd like to ask you some questions about your participation in certain types of work activities and on making decisions on various aspects of household life.		Did you [NAME] participate in [ACTIVITY] in the past 12 months (that is, during the last [one/two] cropping seasons), from [PRESENT MONTH] last year to [PRESENT MONTH] this year?	When decisions are made regarding [ACTIVITY], who is it that normally takes the decision?<br><br>**ENTER UP TO THREE (3) MEMBER IDs**<br><br>**IF RESPONSE IS MEMBER ID (SELF) ONLY → *G2.05***<br><br>**OTHER CODES:**<br>NON-HH MEMBER.......94<br>NOT APPLICABLE.......98 → ***NEXT ACTIVITY***			How much input did you have in making decisions about [ACTIVITY]?<br><br>**USE CODE G2↓**	To what extent do you feel you can participate in decisions regarding [ACTIVITY] if you want(ed) to?<br><br>**CIRCLE ONE**	To what extent are you able to access information that you feel is important for making informed decisions regarding [ACTIVITY]?<br><br>**CIRCLE ONE**	How much input did you have in decisions about how to use income generated from [ACTIVITY]?<br><br>**USE CODE G2↓**	How much input did you have in decisions about how much of the outputs of [ACTIVITY] to keep for consumption at home rather than selling?<br><br>**USE CODE G2↓**
**ACTIVITY**		**G2.01**	**G2.02**			**G2.03**	**G2.04**	**G2.05**	**G2.06**	**G2.07**
			**ID #1**	**ID #2**	**ID #3**					
**A**	Staple grain farming and processing of the harvest: grains that are grown primarily for food consumption (rice, maize, wheat, millet)	YES......1<br>NO.......0 → ***ACTIVITY B***					NOT AT ALL...........1<br>SMALL EXTENT....2<br>MEDIUM EXTENT.3<br>TO A HIGH EXTENT 4	NOT AT ALL...........1<br>SMALL EXTENT.....2<br>MEDIUM EXTENT..3<br>TO A HIGH EXTENT 4		
**B**	Horticultural (gardens) or high value crop farming and processing of the harvest	YES......1<br>NO.......0 → ***ACTIVITY C***					NOT AT ALL...........1<br>SMALL EXTENT....2<br>MEDIUM EXTENT.3<br>TO A HIGH EXTENT 4	NOT AT ALL...........1<br>SMALL EXTENT.....2<br>MEDIUM EXTENT..3<br>TO A HIGH EXTENT 4		
**C**	Large llivestock raising (cattle, buffaloes) and processing of milk and/or meat	YES......1<br>NO.......0 → ***ACTIVITY D***					NOT AT ALL.............1<br>SMALL EXTENT........2<br>MEDIUM EXTENT......3<br>TO A HIGH EXTENT...4	NOT AT ALL.............1<br>SMALL EXTENT........2<br>MEDIUM EXTENT......3<br>TO A HIGH EXTENT...4		

DRAFT

D	Small llivestock raising (sheep, goats, pigs) and processing of milk and/or meat	YES......1<br>NO.......0 → ***ACTIVITY E***					NOT AT ALL.............1<br>SMALL EXTENT........2<br>MEDIUM EXTENT......3<br>TO A HIGH EXTENT...4	NOT AT ALL.............1<br>SMALL EXTENT........2<br>MEDIUM EXTENT......3<br>TO A HIGH EXTENT...4		
E	Poultry and other small animals raising (chickens, ducks, turkeys) and processing of eggs and/or meat	YES......1<br>NO.......0 → ***ACTIVITY F***					NOT AT ALL.............1<br>SMALL EXTENT........2<br>MEDIUM EXTENT......3<br>TO A HIGH EXTENT...4	NOT AT ALL.............1<br>SMALL EXTENT........2<br>MEDIUM EXTENT......3<br>TO A HIGH EXTENT...4		
F	Fishpond culture	YES......1<br>NO.......0 → ***ACTIVITY G***					NOT AT ALL...........1<br>SMALL EXTENT....2<br>MEDIUM EXTENT.3<br>TO A HIGH EXTENT 4	NOT AT ALL...........1<br>SMALL EXTENT.....2<br>MEDIUM EXTENT..3<br>TO A HIGH EXTENT 4		
G	Non-farm economic activities (running a small business, self-employment, buy-and-sell)	YES......1<br>NO.......0 → ***ACTIVITY H***					NOT AT ALL...........1<br>SMALL EXTENT....2<br>MEDIUM EXTENT.3<br>TO A HIGH EXTENT 4	NOT AT ALL...........1<br>SMALL EXTENT.....2<br>MEDIUM EXTENT..3<br>TO A HIGH EXTENT 4		
H	Wage and salary employment (work that is paid for in cash or in-kind, including both agriculture and other wage work)	YES......1<br>NO.......0 → ***ACTIVITY I***					NOT AT ALL...........1<br>SMALL EXTENT....2<br>MEDIUM EXTENT.3<br>TO A HIGH EXTENT 4	NOT AT ALL...........1<br>SMALL EXTENT.....2<br>MEDIUM EXTENT..3<br>TO A HIGH EXTENT 4		
I	Large, occasional household purchases (bicycles, land, transport vehicles)	YES......1<br>NO.......0 → ***ACTIVITY J***					NOT AT ALL...........1<br>SMALL EXTENT....2<br>MEDIUM EXTENT.3<br>TO A HIGH EXTENT 4	NOT AT ALL...........1<br>SMALL EXTENT.....2<br>MEDIUM EXTENT..3<br>TO A HIGH EXTENT 4		
J	Routine household purchases (food for daily consumption or other household needs)	YES......1<br>NO.......0 .... → *ACTIVITY K*					NOT AT ALL...........1<br>SMALL EXTENT....2<br>MEDIUM EXTENT.3<br>TO A HIGH EXTENT 4	NOT AT ALL...........1<br>SMALL EXTENT.....2<br>MEDIUM EXTENT..3<br>TO A HIGH EXTENT 4		
K	Obtaining agricultural inputs (e.g. fertilizers, feed) and services (veterinary, advisory)	YES......1<br>NO.......0 → G3.08					NOT AT ALL...........1<br>SMALL EXTENT....2<br>MEDIUM EXTENT.3<br>TO A HIGH EXTENT 4	NOT AT ALL...........1<br>SMALL EXTENT.....2<br>MEDIUM EXTENT..3<br>TO A HIGH EXTENT 4		

DRAFT

CODE G2
LITTLE TO NO INPUT IN DECISIONS.......................... 1<br>INPUT INTO SOME DECISIONS .... 2<br>INPUT INTO MOST OR ALL DECISIONS..................... 3<br>NOT APPICABLE / NO DECISION MADE 4

Now I'd like to ask you some questions about your participation in certain types of work activities and on making decisions on various aspects of household life. The questions cover a range of activities, with special emphasis on livestock raising.		
**QUESTION**		**RESPONSE**
G2.08. Which of the following species or breed types of livestock are raised in your household	CIRCLE ALL APPLICABLE	LARGE RUMINANT (DAIRY LOCAL) …1<br>LARGE RUMINANT (DAIRY IMPROVED BREEDS) …2<br>LARGE RUMINANT (BEEF OR MIXED LOCAL) …3<br>LARGE RUMINANT (BEEF OR MIXED IMPROVED BREEDS)…4<br>SMALL RUMINANT (SHEEP, GOAT LOCAL)….5<br>SMALL RUMINANT (SHEEP, GOAT IMPROVED BREEDS)…6<br>POULTRY (LOCAL)….7<br>POULTRY (IMPROVED BREEDS)….8<br>PIGS (LOCAL)…9<br>PIGS (IMPROVED BREEDS)…..10<br>CAMELS….11<br>OTHERS SPECIFY….
G2.09 Please select the one most important species for your household livelihood	CIRCLE ONE	LARGE RUMINANT (DAIRY LOCAL) …1<br>LARGE RUMINANT (DAIRY IMPROVED BREEDS) …2<br>LARGE RUMINANT (BEEF OR MIXED LOCAL) …3<br>LARGE RUMINANT (BEEF OR MIXED IMPROVED BREEDS)…4<br>SMALL RUMINANT (SHEEP, GOAT LOCAL)….5<br>SMALL RUMINANT (SHEEP, GOAT IMPROVED BREEDS)…6<br>POULTRY (LOCAL)….7<br>POULTRY (IMPROVED BREEDS)….8<br>PIGS (LOCAL)…9<br>PIGS (IMPROVED BREEDS)…..10<br>CAMELS….11<br>OTHERS SPECIFY….
G2.09a: Reason why it is most important to your household's livelihood:		
G2.10 Please select the most important species for your own livelihood	CIRCLE ONE<br><br>**(CAN BE THE SAME AS FOR G3.09)**	LARGE RUMINANT (DAIRY LOCAL) …1<br>LARGE RUMINANT (DAIRY IMPROVED BREEDS) …2<br>LARGE RUMINANT (BEEF OR MIXED LOCAL) …3<br>LARGE RUMINANT (BEEF OR MIXED IMPROVED BREEDS)…4<br>SMALL RUMINANT (SHEEP, GOAT LOCAL)….5<br>SMALL RUMINANT (SHEEP, GOAT IMPROVED BREEDS)…6<br>POULTRY (LOCAL)….7<br>POULTRY (IMPROVED BREEDS)….8

DRAFT

		PIGS (LOCAL)…9<br>PIGS (IMPROVED BREEDS)…..10<br>CAMELS….11<br>OTHERS SPECIFY….
G2.10a: Reason why it is most important to your own livelihood:		
G2.11 Is there any other species that you feel is important for your livelihood that your household does NOT keep?	CIRCLE ALL APPLICABLE	LARGE RUMINANT (DAIRY LOCAL) …1<br>LARGE RUMINANT (DAIRY IMPROVED BREEDS) …2<br>LARGE RUMINANT (BEEF OR MIXED LOCAL) …3<br>LARGE RUMINANT (BEEF OR MIXED IMPROVED BREEDS)…4<br>SMALL RUMINANT (SHEEP, GOAT LOCAL)….5<br>SMALL RUMINANT (SHEEP, GOAT IMPROVED BREEDS)…6<br>POULTRY (LOCAL)….7<br>POULTRY (IMPROVED BREEDS)….8<br>PIGS (LOCAL)…9<br>PIGS (IMPROVED BREEDS)…..10<br>CAMELS….11<br>OTHERS SPECIFY….
G2.11a: REASON WHY YOUR HOUSEHOLD IS NOT KEEPING THIS LIVESTOCK SPECIES?		

DRAFT

QUESTIONS G2.12–G2.20 (A–E) SHOULD BE ASKED FOR EACH OF THE TWO SPECIES OF LIVESTOCK INDICATED BY QUESTION G2.09 AND G2.10

DRAFT

		Did you [NAME] participate in [ACTIVITY] in the past 12 months (that is during the last [one/two] cropping seasons), from [PRESENT MONTH] last year to [PRESENT MONTH] this year?	How regularly do you perform this activity? CODE FREQUENCY	How many hours do you spend on [ACTIVITY]	When decisions are made regarding [ACTIVITY], who is it that normally takes the decision?<br>**ENTER UP TO THREE (3) MEMBER IDs**<br>**IF RESPONSE IS MEMBER ID (SELF) ONLY -> G2.16**<br>**OTHER CODES:**<br>NON-HH MEMBER…94<br>NOT APPLICABLE…98<br>➔ **NEXT DECISION**			How much input did you have in making decisions about [ACTIVITY] if you want(ed) to?<br>**USE CODE G2** ↓	To what extent do you feel you can participate in decisions regarding [ACTIVITY] if you want(ed) to?<br>**CIRCLE ONE**	Who would you prefer made the decisions about [ACTIVITY]?<br>ENTER UP TO THREE (3) MEMBER IDs<br>**OTHER CODES:**<br>NON-HH MEMBER……94<br>NOT APPLICABLE…….98			To what extent are you able to access information that you feel is important for making informed decisions regarding [ACTIVITY]?<br>**CIRCLE ONE**	How much input did you have in decisions about how much of the outputs of [ACTIVITY] to keep for consumption at home rather than selling?<br>**USE CODE G2** ↓	How much input did you have in decisions about how to use income generated from [ACTIVITY]?<br>**USE CODE G2** ↓
	**ACTIVITY [ANIMAL]**	**G2.12**	**G2.13**	**G2.13a**	**G2.14**			**G2.15**	**G2.16**	**G2.17**			**G2.18**	**G2.19**	**G2.20**
					**ID 1**	**ID2**	**ID3**			**ID 1**	**ID2**	**ID3**			
**A**	Animal feeding (collect, purchase, prepare or bring feed to animals)	YES…1<br>NO…2 -> **ACTIVITY B**							NOT AT ALL............................ 1<br>SMALL EXTENT .... 2<br>MEDIUM EXTENT .... 3<br>TO A HIGH EXTENT…………. 4				NOT AT ALL .... 1<br>SMALL EXTENT .... 2<br>MEDIUM EXTENT .... 3<br>TO A HIGH EXTENT…………. 4		
B	Animal watering (collect or bring water to animal)	YES…1<br>NO…2 -> **ACTIVITY C**							NOT AT ALL............................ 1<br>SMALL EXTENT .... 2<br>MEDIUM EXTENT .... 3<br>TO A HIGH EXTENT…………. 4				NOT AT ALL .... 1<br>SMALL EXTENT .... 2<br>MEDIUM EXTENT .... 3<br>TO A HIGH EXTENT…………. 4		
C	Animal grazing (taking animal out of the farm for grazing)	YES…1<br>NO…2 -> **ACTIVITY D**							NOT AT ALL............................ 1<br>SMALL EXTENT .... 2<br>MEDIUM EXTENT .... 3<br>TO A HIGH EXTENT…………. 4				NOT AT ALL .... 1<br>SMALL EXTENT .... 2<br>MEDIUM EXTENT .... 3<br>TO A HIGH EXTENT…………. 4		

D	Check animal health	YES...1<br>NO...2 -><br>**ACTIVITY E**							NOT AT ALL............................ 1<br>SMALL EXTENT .... 2<br>MEDIUM EXTENT .... 3<br>TO A HIGH EXTENT............ 4				NOT AT ALL .... 1<br>SMALL EXTENT .... 2<br>MEDIUM EXTENT .... 3<br>TO A HIGH EXTENT............ 4		
E	Carry out disease preventive measures (e.g. spraying, deworming, or taking animals to dip)	YES...1<br>NO...2 -><br>**ACTIVITY F**							NOT AT ALL............................ 1<br>SMALL EXTENT .... 2<br>MEDIUM EXTENT .... 3<br>TO A HIGH EXTENT............ 4				NOT AT ALL .... 1<br>SMALL EXTENT .... 2<br>MEDIUM EXTENT .... 3<br>TO A HIGH EXTENT............ 4		
F	Carry out curative measures (e.g. give medicines to heal sick animals)	YES...1<br>NO...2 -><br>**ACTIVITY G**							NOT AT ALL............................ 1<br>SMALL EXTENT .... 2<br>MEDIUM EXTENT .... 3<br>TO A HIGH EXTENT............ 4				NOT AT ALL .... 1<br>SMALL EXTENT .... 2<br>MEDIUM EXTENT .... 3<br>TO A HIGH EXTENT............ 4		
G	Milking animals	YES...1<br>NO...2 -><br>**ACTIVITY H**							NOT AT ALL............................ 1<br>SMALL EXTENT .... 2<br>MEDIUM EXTENT .... 3<br>TO A HIGH EXTENT............ 4				NOT AT ALL .... 1<br>SMALL EXTENT .... 2<br>MEDIUM EXTENT .... 3<br>TO A HIGH EXTENT............ 4		
H	Selling milk/eggs	YES...1<br>NO...2 -><br>**ACTIVITY I**							NOT AT ALL............................ 1<br>SMALL EXTENT .... 2<br>MEDIUM EXTENT .... 3<br>TO A HIGH EXTENT............ 4				NOT AT ALL .... 1<br>SMALL EXTENT .... 2<br>MEDIUM EXTENT .... 3<br>TO A HIGH EXTENT............ 4		
I	Cleaning animals, shelter or utensils	YES...1<br>NO...2 -><br>**ACTIVITY J**							NOT AT ALL............................ 1<br>SMALL EXTENT .... 2<br>MEDIUM EXTENT .... 3<br>TO A HIGH EXTENT............ 4				NOT AT ALL .... 1<br>SMALL EXTENT .... 2<br>MEDIUM EXTENT .... 3<br>TO A HIGH EXTENT............ 4		
J	Slaughter animals	YES...1<br>NO...2 -><br>**ACTIVITY K**							NOT AT ALL............................ 1<br>SMALL EXTENT .... 2<br>MEDIUM EXTENT .... 3<br>TO A HIGH EXTENT............ 4				NOT AT ALL .... 1<br>SMALL EXTENT .... 2<br>MEDIUM EXTENT .... 3<br>TO A HIGH EXTENT............ 4		
K	Prepare animal meat, eggs, milk into food	YES...1<br>NO...2 -><br>**ACTIVITY L**							NOT AT ALL............................ 1<br>SMALL EXTENT .... 2<br>MEDIUM EXTENT .... 3<br>TO A HIGH EXTENT............ 4				NOT AT ALL .... 1<br>SMALL EXTENT .... 2<br>MEDIUM EXTENT .... 3<br>TO A HIGH EXTENT............ 4		
L	Breeding animals in own flock (1 choose female and male	YES...1<br>NO...2 -><br>**ACTIVITY M**							NOT AT ALL............................ 1<br>SMALL EXTENT .... 2<br>MEDIUM EXTENT .... 3<br>TO A HIGH EXTENT............ 4				NOT AT ALL .... 1<br>SMALL EXTENT .... 2<br>MEDIUM EXTENT .... 3<br>TO A HIGH EXTENT............ 4		

DRAFT

	animals to parent the next generation; 2 nurture the selected parents through better care; 3 separate males and females and arrange their mating at appropriate times)														
M	Arrange for artificial insemination (1. contact the AI provider; 2 choose the animals to parent the next generation; 3 arrange to receive AI service)	YES...1<br>NO...2 -><br>**ACTIVITY N**							NOT AT ALL.............................. 1<br>SMALL EXTENT .... 2<br>MEDIUM EXTENT .... 3<br>TO A HIGH EXTENT............. 4				NOT AT ALL .... 1<br>SMALL EXTENT .... 2<br>MEDIUM EXTENT .... 3<br>TO A HIGH EXTENT............. 4		
N	Receiving sire service (1. look for others to provide male animal for breeding; 2 choose the animals to parent the next generation; 3 arrange to receive sire service)	YES...1<br>NO...2 -><br>**ACTIVITY O**							NOT AT ALL.............................. 1<br>SMALL EXTENT .... 2<br>MEDIUM EXTENT .... 3<br>TO A HIGH EXTENT............. 4				NOT AT ALL .... 1<br>SMALL EXTENT .... 2<br>MEDIUM EXTENT .... 3<br>TO A HIGH EXTENT............. 4		
O	Deciding how much	YES...1<br>NO...2 ->							NOT AT ALL.............................. 1<br>SMALL EXTENT 2				NOT AT ALL .... 1<br>SMALL EXTENT 2		

DRAFT

	product from [ANIMAL] to put aside for household consumption	**ACTIVITY P**							MEDIUM EXTENT .... 3<br>TO A HIGH EXTENT............ 4				MEDIUM EXTENT .... 3<br>TO A HIGH EXTENT............ 4		
P	Marketing of live animals and any animal products (except milk and eggs)	YES...1<br>NO...2 -><br>**ACTIVITY Q**							NOT AT ALL.......................... 1<br>SMALL EXTENT .... 2<br>MEDIUM EXTENT .... 3<br>TO A HIGH EXTENT............ 4				NOT AT ALL .... 1<br>SMALL EXTENT .... 2<br>MEDIUM EXTENT .... 3<br>TO A HIGH EXTENT............ 4		
Q	Selecting which species and which breeds to rear	YES...1<br>NO...2 -><br>**ACTIVITY R**							NOT AT ALL.......................... 1<br>SMALL EXTENT .... 2<br>MEDIUM EXTENT .... 3<br>TO A HIGH EXTENT............ 4				NOT AT ALL .... 1<br>SMALL EXTENT .... 2<br>MEDIUM EXTENT .... 3<br>TO A HIGH EXTENT............ 4		
R	Sharing livestock workload among household members	YES...1<br>NO...2 -><br>**ACTIVITY S**							NOT AT ALL.......................... 1<br>SMALL EXTENT .... 2<br>MEDIUM EXTENT .... 3<br>TO A HIGH EXTENT............ 4						
S	Providing this livestock as collateral to access credit	YES...1<br>NO...2 -><br>**ACTIVITY T**							NOT AT ALL.......................... 1<br>SMALL EXTENT .... 2<br>MEDIUM EXTENT .... 3<br>TO A HIGH EXTENT............ 4						
T	Using dung from livestock or using livestock as draft power	YES...1<br>NO...2 -><br>**ACTIVITY G3**							NOT AT ALL.......................... 1<br>SMALL EXTENT .... 2<br>MEDIUM EXTENT .... 3<br>TO A HIGH EXTENT............ 4						

**CODE G2**	**CODE G2.13**	**CODE G2.13a**
LITTLE TO NO INPUT IN DECISIONS .... 1<br>INPUT INTO SOME DECISIONS .... 2<br>INPUT INTO MOST OR ALL DECISIONS .... 3<br>NOT APPLICABLE / NO DECISION MADE 98	Daily ……………………….....1<br>Two times a week ……..........2<br>One time a week ….…............3<br>Two times a month …….........4<br>One time a month ……..........5<br>Every three months ……........6<br>Two times a year ……...........7<br>One time a year …….............8<br>Other (specify) ……..............9	Above 40 hours …0<br>20-40 hours ……..1<br>10-20 hours……...2<br>5-10 hours……….3<br>0-5 hours……......4<br>None……………..5

HOUSEHOLD ID						
RESPONDENT ID						

MODULE G3(A): ACCESS TO PRODUCTIVE CAPITAL

DRAFT

Now I'd like to ask you specifically about your household's land.

QUESTION		RESPONSE		
**G3.01.** Does anyone in your household currently own or cultivate land?		YES........1<br>NO.........0 → ***G3.06, ITEM A***		
**G3.01a:** How much land does your household owned (Acres)?				
**G3.01b:** How much land is cultivated (Acres), from the Total land owned?				
**G3.01c:** How much of rented-in land is cultivated (Acres)?				
**G3.02.** Who generally makes decisions about what to plant on this land?<br>And what to do with the output from this land?	**ENTER UP TO THREE (3) MEMBER IDs**<br>**OTHER CODES:**<br>NON-HH MEMBER .... 94<br>NOT APPLICABLE 98	**ID #1**	**ID #2**	**ID #3**
**G3.03.** Do you [NAME] solely or jointly cultivate any land?	**CIRCLE <u>ONE</u>**	YES, SOLELY ....1<br>YES, JOINTLY..2<br>YES, SOLELY AND JOINTLY.................3<br>NO 4		
**G3.04.** Who generally makes decisions about what to plant on the land that you yourself cultivate?<br>And what to do with the output from this land?	**ENTER UP TO THREE (3) MEMBER IDs**<br>**OTHER CODES:**<br>NON-HH MEMBER .... 94<br>NOT APPLICABLE 98	**ID #1**	**ID #2**	**ID #3**
**G3.05.** Do you own any of the land owned or cultivated by your household?	**CIRCLE <u>ONE</u>**	YES, SOLELY ....1<br>YES, JOINTLY..2<br>YES, SOLELY AND JOINTLY.................3<br>NO 4		

DRAFT

Now I'd like to ask you about a number of items that could be used to generate income.		Does anyone in your household currently have any [ITEM]?	Do you [NAME] own any [ITEM]?<br><br>**CIRCLE ONE**	Total number owned	Number owned solely	Number owned jointly	For assets you own (solely), which of the following can you do on your own, without consultation?<br><br>**CIRCLE ALL APPLICABLE**	With whom do you co-own [ITEM]?<br><br>**CIRCLE ALL APPLICABLE**	For assets you own (jointly) with someone else, which of the following can you do on your own, without consultation?<br><br>**CIRCLE ALL APPLICABLE**:	For all assets that you own (solely or jointly), which of the following can your spouse do on his/her own, without consulting you?<br><br>**CIRCLE ALL APPLICABLE**
**ITEM**		**G3.06**	**G3.07**	**G3.07a**	**G3.07b**	**G3.07c**	**G3.08**	**G3.09**	**G3.10**	**G3.11**
**A**	Large ruminant (dairy)	YES........1<br>NO.........0 → ***ITEM B***	YES, SOLELY.........................1<br>YES, JOINTLY.......................2<br>→ ***G3.09***<br>YES, SOLELY AND JOINTLY.....3 → ***G3.09***<br>NO.......................................4 → ***ITEM B***				GIVE AS GIFT ....1<br>SELL ....2<br>LOAN TO SOMEONE ELSE 3<br>PLEDGE AS COLLATERAL.4<br>LOOK AFTER LIVESTOCK.... 5<br>SLAUGTHER .... 6<br>→ ***G3.11***	SPOUSE.......................1<br>OTHER HH MEMBER..2<br>NON HH-MEMBER 3		GIVE AS GIFT ....1<br>SELL ....2<br>LOAN TO SOMEONE ELSE ....3<br>PLEDGE AS COLLATERAL................4<br>LOOK AFTER LIVESTOCK.... 5<br>SLAUGTHER 6
**B**	Large ruminant (beef or mixed)	YES........1<br>NO.........0 → ***ITEM C***	YES, SOLELY.........................1<br>YES, JOINTLY.......................2<br>→ ***G3.09***<br>YES, SOLELY AND JOINTLY.....3 → ***G3.09***<br>NO.......................................4 → ***ITEM C***				GIVE AS GIFT ....1<br>SELL ....2<br>LOAN TO SOMEONE ELSE 3<br>PLEDGE AS COLLATERAL.4<br>LOOK AFTER LIVESTOCK.... 5<br>SLAUGTHER .... 6<br>→ ***G3.11***	SPOUSE.......................1<br>OTHER HH MEMBER..2<br>NON HH-MEMBER 3		GIVE AS GIFT ....1<br>SELL ....2<br>LOAN TO SOMEONE ELSE ....3<br>PLEDGE AS COLLATERAL................4<br>LOOK AFTER LIVESTOCK.... 5<br>SLAUGTHER 6
**C**	Small ruminant (Sheep, goats)	YES........1<br>NO.........0 → ***ITEM D***	YES, SOLELY.........................1<br>YES, JOINTLY.......................2<br>→ ***G3.09***<br>YES, SOLELY AND JOINTLY.....3 → ***G3.09***<br>NO.......................................4 → ***ITEM D***				GIVE AS GIFT ....1<br>SELL ....2<br>LOAN TO SOMEONE ELSE 3<br>PLEDGE AS COLLATERAL.4<br>LOOK AFTER LIVESTOCK.... 5<br>SLAUGTHER .... 6<br>→ ***G3.11***	SPOUSE.......................1<br>OTHER HH MEMBER..2<br>NON HH-MEMBER 3		GIVE AS GIFT ....1<br>SELL ....2<br>LOAN TO SOMEONE ELSE ....3<br>PLEDGE AS COLLATERAL................4<br>LOOK AFTER LIVESTOCK.... 5<br>SLAUGTHER 6

**D**	Poultry	YES........1<br>NO.........0 → ***ITEM E***	YES, SOLELY.........................1<br>YES, JOINTLY.......................2 → ***G3.09***<br>YES, SOLELY AND JOINTLY.....3 → ***G3.09***<br>NO.................................<br>.......4 → ***ITEM E***				GIVE AS GIFT ....1<br>SELL ....2<br>LOAN TO SOMEONE ELSE 3<br>PLEDGE AS COLLATERAL.4<br>LOOK AFTER LIVESTOCK.... 5<br>SLAUGHTER .... 6<br>→ ***G3.11***	SPOUSE.......................1<br>OTHER HH MEMBER..2<br>NON HH-MEMBER 3		GIVE AS GIFT ....1<br>SELL ....2<br>LOAN TO SOMEONE ELSE ....3<br>PLEDGE AS COLLATERAL................4<br>LOOK AFTER LIVESTOCK.... 5<br>SLAUGTHER 6
**E**	Pigs	YES........1<br>NO.........0 → ***ITEM F***	YES, SOLELY.........................1<br>YES, JOINTLY.......................2 → ***G3.09***<br>YES, SOLELY AND JOINTLY.....3 → ***G3.09***<br>NO.................................<br>.......4 → ***ITEM F***					SPOUSE.......................1<br>OTHER HH MEMBER..2<br>NON HH-MEMBER 3		GIVE AS GIFT ....1<br>SELL ....2<br>LOAN TO SOMEONE ELSE ....3<br>PLEDGE AS COLLATERAL................4<br>LOOK AFTER LIVESTOCK.... 5<br>SLAUGTHER 6
**F**	Fish pond or fishing equipment	YES........1<br>NO.........0 → ***ITEM G***	YES, SOLELY......................1<br>YES, JOINTLY.....................2<br>YES, SOLELY AND JOINTLY ....3<br>NO..4							
**G**	Non-mechanized farm equipment (hand tools, animal-drawn plough)	YES........1<br>NO.........0 → ***ITEM H***	YES, SOLELY......................1<br>YES, JOINTLY.....................2<br>YES, SOLELY AND JOINTLY ....3<br>NO..4							
**H**	Mechanized farm equipment (tractor-plough, power tiller, treadle pump)	YES........1<br>NO.........0 → ***ITEM I***	YES, SOLELY......................1<br>YES, JOINTLY.....................2<br>YES, SOLELY AND JOINTLY ....3<br>NO..4							

DRAFT

**I**	Non-farm business equipment (solar panels used for recharging, sewing machine, brewing equipment, fryers)	YES........1<br>NO.........0 →<br>***ITEM J***	YES, SOLELY......................1<br>YES, JOINTLY.....................2<br>YES, SOLELY AND<br>JOINTLY ....3<br>NO..4							
**J**	House or building	YES........1<br>NO.........0 →<br>***ITEM J***	YES, SOLELY......................1<br>YES, JOINTLY.....................2<br>YES, SOLELY AND<br>JOINTLY ....3<br>NO..4							
**K**	Large consumer durables (refrigerator, TV, sofa)	YES........1<br>NO.........0 →<br>***ITEM K***	YES, SOLELY......................1<br>YES, JOINTLY.....................2<br>YES, SOLELY AND<br>JOINTLY ....3<br>NO..4							
**L**	Small consumer durables (radio, cookware)	YES........1<br>NO.........0 →<br>***ITEM L***	YES, SOLELY......................1<br>YES, JOINTLY.....................2<br>YES, SOLELY AND<br>JOINTLY ....3<br>NO..4							
**M**	Cell phone	YES........1<br>NO.........0 →<br>***ITEM M***	YES, SOLELY......................1<br>YES, JOINTLY.....................2<br>YES, SOLELY AND<br>JOINTLY ....3<br>NO..4							

**N**	Other land not used for agricultural purposes (pieces/plots, residential or commercial land)	YES........1<br>NO.........0 → ***ITEM N***	YES, SOLELY......................1<br>YES, JOINTLY.....................2<br>YES, SOLELY AND JOINTLY ....3<br>NO.......................................4								
**O**	Means of transportation (bicycle, motorcycle, car)	YES........1<br>NO.........0 → ***MODULE G3(B)***	YES, SOLELY......................1<br>YES, JOINTLY.....................2<br>YES, SOLELY AND JOINTLY ....3<br>NO.......................................4								

DRAFT

MODULE G3(B): ACCESS TO FINANCIAL SERVICES

DRAFT

Next I'd like to ask about your household's experience with borrowing money or other items (in-kind) in the past 12 months.		Would you or anyone in your household be able to take a loan or borrow cash/in-kind from [SOURCE] if you wanted to?	Has anyone in your household taken any loans or borrowed cash/in-kind from [SOURCE] in the past 12 months?<br><br>**CIRCLE ONE**	Who made the decision to borrow from [SOURCE] most of the time?<br><br>**ENTER UP TO THREE (3) MEMBER IDs**<br><br>**OTHER CODES:**<br>NON-HH MEMBER.......94<br>NOT APPLICABLE.......98			Do you think the decision to borrow [ITEM] was a good decision?<br><br>YES.........1<br>NO..........0	Who makes the decision about what to do with the money or item borrowed from [SOURCE] most of the time?<br><br>**ENTER UP TO THREE (3) MEMBER IDs**<br><br>**OTHER CODES:**<br>NON-HH MEMBER.......94<br>NOT APPLICABLE.......98			Who is responsible for repaying the money or item borrowed from [SOURCE]?<br><br>**ENTER UP TO THREE (3) MEMBER IDs**<br><br>**OTHER CODES:**<br>NON-HH MEMBER.......94<br>NOT APPLICABLE.......98		
**LENDING SOURCES**		**G3.08**	**G3.09**	**G3.10**			**G3.11**	**G3.12**			**G3.13**		
				**ID #1**	**ID #2**	**ID #3**		**ID #1**	**ID #2**	**ID #3**	**ID #1**	**ID #2**	**ID #3**
**A**	Non-governmental organization (NGO)	YES..........1<br>NO..........0 → ***SOURCE B***<br>MAYBE.....3	YES, CASH............................1<br>YES, IN-KIND ....2<br>YES, CASH AND IN-KIND ....3<br>NO..4 → ***SOURCE B***<br>DON'T KNOW.....................88										
**B**	Formal lender (bank/financial institution)	YES..........1<br>NO..........0 → ***SOURCE C***<br>MAYBE.....3	YES, CASH............................1<br>YES, IN-KIND ....2<br>YES, CASH AND IN-KIND ....3<br>NO..4 → ***SOURCE C***<br>DON'T KNOW .88										
**C**	Informal lender	YES..........1<br>NO..........0 → ***SOURCE D***<br>MAYBE.....3	YES, CASH............................1<br>YES, IN-KIND ....2<br>YES, CASH AND IN-KIND ....3<br>NO..4 → ***SOURCE D***<br>DON'T KNOW .88										
**D**	Friends or relatives	YES..........1<br>NO..........0 → ***SOURCE E***<br>MAYBE.....3	YES, CASH............................1<br>YES, IN-KIND ....2<br>YES, CASH AND IN-KIND ....3<br>NO..4 → ***SOURCE E***<br>DON'T KNOW..88										
**E**	Group based micro-finance or lending including VSLAs / SACCOs	YES..........1<br>NO..........0 → ***SOURCE F***<br>MAYBE.....3	YES, CASH............................1<br>YES, IN-KIND ....2<br>YES, CASH AND IN-KIND ....3<br>NO..4 → ***SOURCE F***<br>DON'T KNOW..88										
**F**	Informal credit / savings groups (. e.g., merry-go-rounds, tontines,	YES..........1<br>NO..........0 → ***G3.13***<br>MAYBE.....3	YES, CASH............................1<br>YES, IN-KIND ....2<br>YES, CASH AND IN-KIND ....3<br>NO..4 → ***G3.13***										

	funeral societies, etc.)		DON'T KNOW......................88										

**G3.14**	An account can be used to save money, to make or receive payments, or to receive wages or financial help. Do you, either by yourself or together with someone else, currently have an account at any of the following places: a bank or other formal institution (e.g., post office)?	YES.........1<br>NO.........0<br>DON'T KNOW.........88

DRAFT

HOUSEHOLD ID						
				RESPONDENT ID		

MODULE G4: TIME ALLOCATION

G4.01: PLEASE RECORD A LOG OF THE ACTIVITIES FOR THE INDIVIDUAL IN THE LAST COMPLETE 24 HOURS (STARTING YESTERDAY MORNING AT 4 AM, FINISHING 3:59 AM OF THE CURRENT DAY). THE TIME INTERVALS ARE MARKED IN 15 MIN INTERVALS. MARK ONE PRIMARY ACTIVITY FOR EACH TIME PERIOD BY ENTERING THE CORRESPONDING ACTIVITY CODE IN THE BOX.
G4.02: CHECK THE BOX BELOW IF THE RESPONDENT WAS CARING FOR CHILDREN WHILE PERFORMING EACH ACTIVITY.

Now I'd like to ask you about how you spent your time during the past 24 hours. We'll begin from yesterday morning and continue through to this morning. This will be a detailed accounting. I'm interested in everything you did (i.e. resting, eating, personal care, work inside and outside the home, caring for children, cooking, shopping, socializing, etc.), even if it didn't take you much time. I'm particularly interested in agricultural activities such as farming, gardening, and livestock raising whether in the field or on the homestead. I'm also interested in how much time you spent caring for children, especially if it happened while you did some other activity (e.g., collecting water while carrying a child or cooking while watching after a sleeping child).

		Night		Morning	Day								
		4:00	5:00	6:00	7:00	8:00	9:00	10:00	11:00	12:00	13:00	14:00	15:00
**G4.01** Activity **(WRITE ACTIVITY CODE)**													
**G4.02** Did you also care for children?	YES......CHECK BOX<br>NO.....LEAVE BLANK	☐☐☐☐	☐☐☐☐	☐☐☐☐	☐☐☐☐	☐☐☐☐	☐☐☐☐	☐☐☐☐	☐☐☐☐	☐☐☐☐	☐☐☐☐	☐☐☐☐	☐☐☐☐
		Day		Evening	Night								
		16:00	17:00	18:00	19:00	20:00	21:00	22:00	23:00	24:00	1:00	2:00	3:00
**G4.01** Activity **(WRITE ACTIVITY CODE)**													
**G4.02** Did you also care for children?	YES......CHECK BOX<br>NO.....LEAVE BLANK	☐☐☐☐	☐☐☐☐	☐☐☐☐	☐☐☐☐	☐☐☐☐	☐☐☐☐	☐☐☐☐	☐☐☐☐	☐☐☐☐	☐☐☐☐	☐☐☐☐	☐☐☐☐

ACTIVITY CODES FOR G4.01

A .... Sleeping and resting	H.....Horticultural (gardens) or high value crop farming	N...................... Shopping / getting service (incl. health services)	U ....Exercising
B .... Eating and drinking	I ....Large livestock raising (cattle, buffaloes)	O...Weaving / sewing / textile care	V ....Social activities and hobbies
C.....................Personal care	J ....Small livestock raising (sheep, goats, pigs)	P .... Cooking	W............Religious activities
D.....School (incl. homework)	K....................Poultry and other small animals raising (chickens, ducks, turkeys)	Q............Domestic work (incl. fetching water and collecting fuel)	X ....Other (specify)
E .... Work as employed		R..Caring for children	Y ....Selling milk and other livestock produce
F ....Own business work	L .... Fishpond culture	S ....Caring for adults (sick, elderly)	
G...........Staple grain farming	M ....Commuting (to/from work or school)	T ....Traveling (not for work or school)	
		…………………………..Watching TV/listening to radio/reading	

DRAFT

**G4.03.** In the last 24 hours did you work (at home or outside of the home including chores or other domestic activities) less than usual, about the same as usual, or more than usual?	**FOR <u>FEMALES</u> ONLY: DOES RESPONDENT HAVE A CHILD UNDER 5 YEARS OLD?**	**G4.04.** If you wanted to do something (livelihood-related, training-related, self-care) and could not take your child with you, is there someone who could care for your child in your absence?	**G4.05.** Who? **ENTER UP TO THREE (3) MEMBER IDs**	**ID #1**	**ID #2**	**ID #3**
LESS THAN USUAL .... 3<br>ABOUT THE SAME AS USUAL .... 2<br>MORE THAN USUAL .... 1<br><br>**IF RESPONDENT IS <u>MALE</u> → MODULE G5**	YES.........1 → G4.04<br>NO.......... 0 → MODULE G5	YES..........1 → G4.05<br>NO...........0 → MODULE G5	**OTHER CODES:**<br>NON-HH MEMBER.......94<br>NOT APPLICABLE.......98			

DRAFT

HOUSEHOLD ID						
			RESPONDENT ID			

DRAFT

MODULE G5: GROUP MEMBERSHIP

Now I'm going to ask you about groups in the community. These can be either formal or informal and customary groups.		Is there a [GROUP] in your community?	Are you an active member of this [GROUP]?	Is this group composed of all male or female or mixed-sex [illegible]	To what extent do you feel like you can influence decisions in this [GROUP]?	To what extent does this [GROUP] influence life in the community beyond the group activities?
**GROUP CATEGORIES**		**G5.01**	**G5.02**	**G5.03**	**G5.04**	**G5.05**
**A**	Agricultural / livestock / fisheries producer's group (including marketing groups)	YES....................1<br>NO .... 0 → ***GROUP B***<br>DON'T KNOW 88	YES......1<br>NO........2 → ***GROUP B***	ALL MALE ....1<br>ALL FEMALE.........2<br>MIXED SEX...........3<br>DON'T KNOW 97	NOT AT ALL ....1<br>SMALL EXTENT..........2<br>MEDIUM EXTENT.......3<br>HIGH EXTENT 4	NOT AT ALL..........................1<br>SMALL EXTENT ....2<br>MEDIUM EXTENT ....3<br>HIGH EXTENT......................4
**B**	Water users' group	YES....................1<br>NO .... 0 → ***GROUP C***<br>DON'T KNOW 88	YES......1<br>NO........2 → ***GROUP C***	ALL MALE ....1<br>ALL FEMALE.........2<br>MIXED SEX...........3<br>DON'T KNOW 97	NOT AT ALL ....1<br>SMALL EXTENT..........2<br>MEDIUM EXTENT.......3<br>HIGH EXTENT 4	NOT AT ALL..........................1<br>SMALL EXTENT ....2<br>MEDIUM EXTENT ....3<br>HIGH EXTENT......................4
**C**	Forest users' group	YES....................1<br>NO .... 0 → ***GROUP D***<br>DON'T KNOW 88	YES......1<br>NO........2 → ***GROUP D***	ALL MALE ....1<br>ALL FEMALE.........2<br>MIXED SEX...........3<br>DON'T KNOW 97	NOT AT ALL ....1<br>SMALL EXTENT..........2<br>MEDIUM EXTENT.......3<br>HIGH EXTENT 4	NOT AT ALL..........................1<br>SMALL EXTENT ....2<br>MEDIUM EXTENT ....3<br>HIGH EXTENT......................4
**D**	Credit or microfinance group (including SACCOs / merry-go-rounds / VSLAs)	YES....................1<br>NO .... 0 → ***GROUP E***<br>DON'T KNOW 88	YES......1<br>NO........2 → ***GROUP E***	ALL MALE ....1<br>ALL FEMALE.........2<br>MIXED SEX...........3<br>DON'T KNOW 97	NOT AT ALL ....1<br>SMALL EXTENT..........2<br>MEDIUM EXTENT.......3<br>HIGH EXTENT 4	NOT AT ALL..........................1<br>SMALL EXTENT ....2<br>MEDIUM EXTENT ....3<br>HIGH EXTENT......................4
**E**	Mutual help or insurance group (including burial societies)	YES....................1<br>NO .... 0 → ***GROUP F***<br>DON'T KNOW 88	YES......1<br>NO........2 → ***GROUP F***	ALL MALE ....1<br>ALL FEMALE.........2<br>MIXED SEX...........3<br>DON'T KNOW 97	NOT AT ALL ....1<br>SMALL EXTENT..........2<br>MEDIUM EXTENT.......3<br>HIGH EXTENT 4	NOT AT ALL..........................1<br>SMALL EXTENT ....2<br>MEDIUM EXTENT ....3<br>HIGH EXTENT......................4
**F**	Trade and business association group	YES....................1<br>NO .... 0 → ***GROUP G***<br>DON'T KNOW 88	YES......1<br>NO........2 → ***GROUP G***	ALL MALE ....1<br>ALL FEMALE.........2<br>MIXED SEX...........3<br>DON'T KNOW 97	NOT AT ALL ....1<br>SMALL EXTENT..........2<br>MEDIUM EXTENT.......3<br>HIGH EXTENT 4	NOT AT ALL..........................1<br>SMALL EXTENT ....2<br>MEDIUM EXTENT ....3<br>HIGH EXTENT......................4
**G**	Civic group (improving community) or charitable group (helping others)	YES....................1<br>NO .... 0 → ***GROUP H***<br>DON'T KNOW 88	YES......1<br>NO........2 → ***GROUP H***	ALL MALE ....1<br>ALL FEMALE.........2<br>MIXED SEX...........3<br>DON'T KNOW 97	NOT AT ALL ....1<br>SMALL EXTENT..........2<br>MEDIUM EXTENT.......3<br>HIGH EXTENT 4	NOT AT ALL..........................1<br>SMALL EXTENT ....2<br>MEDIUM EXTENT ....3<br>HIGH EXTENT......................4

H	Religious group	YES....................1<br>NO .... 0<br>DON'T KNOW 88 → ***GROUP I***	YES......1<br>NO........2 → ***GROUP I***	ALL MALE ....1<br>ALL FEMALE.........2<br>MIXED SEX...........3<br>DON'T KNOW 97	NOT AT ALL ....1<br>SMALL EXTENT..........2<br>MEDIUM EXTENT.......3<br>HIGH EXTENT 4	NOT AT ALL.........................1<br>SMALL EXTENT ....2<br>MEDIUM EXTENT ....3<br>HIGH EXTENT......................4
I	Milk/dairy marketing group	YES....................1<br>NO .... 0<br>DON'T KNOW 88 → *GROUP J*	YES......1<br>NO........2 → ***GROUP I***	ALL MALE ....1<br>ALL FEMALE.........2<br>MIXED SEX...........3<br>DON'T KNOW 97	NOT AT ALL ....1<br>SMALL EXTENT..........2<br>MEDIUM EXTENT.......3<br>HIGH EXTENT 4	NOT AT ALL.........................1<br>SMALL EXTENT ....2<br>MEDIUM EXTENT ....3<br>HIGH EXTENT......................4
j	Other (specify):<br>____________________	YES....................1<br>NO .... 0<br>DON'T KNOW 88 → ***MODULE G6***	YES......1<br>NO........2 → ***MODULE G6***	ALL MALE ....1<br>ALL FEMALE.........2<br>MIXED SEX...........3<br>DON'T KNOW 97	NOT AT ALL ....1<br>SMALL EXTENT..........2<br>MEDIUM EXTENT.......3<br>HIGH EXTENT 4	NOT AT ALL.........................1<br>SMALL EXTENT ....2<br>MEDIUM EXTENT ....3<br>HIGH EXTENT......................4

DRAFT

HOUSEHOLD ID						
RESPONDENT ID		

MODULE G6. PHYSICAL MOBILITY

		During the past 12 months, how often do you generally go to [PLACE]?<br><br>**USE CODE G6.01**	Who usually decides whether you can go to [PLACE]?<br><br>**ENTER UP TO THREE (3) MEMBER IDs**<br><br>**IF RESPONSE IS MEMBER ID (SELF) ONLY → NEXT PLACE**<br><br>**OTHER CODES:**<br>NON-HH MEMBER.......94<br>NOT APPLICABLE.......98			Were you ever prevented from going to [PLACE] when you wanted to?	If you wanted to go to [PLACE], but couldn't, why not?<br><br>**USE G6.04. RESPONSE CODES. LIST ALL CODES APPLICABLE.**<br><br>**DO NOT READ RESPONS OPTONS ALOUD. LISTEN TO RESPONDENT AND SELECT APPROPRIATE CODE.**	Does your spouse/partner object to you going alone to [PLACE]?	Under what circumstances would this person NOT object to your going to [PLACE] alone?<br><br>**CIRCLE ALL APPLICABLE**	Do these objections prevent you from going alone to [PLACE]?
**PLACE**		**G6.01**	**G6.02 WOMAN ONLY**<br>**ID #1 ID #2 ID #3**			**G6.03 WOMAN ONLY**	**G6.04 WOMAN ONLY**	**G6.05 WOMAN ONLY**	**G6.06 WOMAN ONLY**	**G6.07 WOMAN ONLY**
**A**	Urban center					YES......1<br>NO........0→ ***G6.05***		YES......1<br>NO........0 → PLACE B	IF I HAVE COMPANY (RELATIVES, CHILDREN)...............................1<br>IF I CAN ARRANGE MY OWN EXPENSES (FOR TRANSPORT).............2<br>IF I FOLLOW PURDAH / DRESS ACCEPTABLY................................3<br>OTHER (SPECIFY)..4<br>UNDER NO CIRCUMSTANCES WOULD I BE ALLOWED TO GO...........5 → ***PLACE B***	YES......1<br>NO........0
**B**	Market / haat / bazaar					YES......1<br>NO........0→ ***G6.05***		YES......1<br>NO........0 → PLACE C	IF I HAVE COMPANY (RELATIVES, CHILDREN)...............................1<br>IF I CAN ARRANGE MY OWN EXPENSES (FOR TRANSPORT).............2<br>IF I FOLLOW PURDAH / DRESS ACCEPTABLY................................3<br>OTHER (SPECIFY)..4<br>UNDER NO CIRCUMSTANCES WOULD I BE ALLOWED TO GO...........5 → ***PLACE C***	YES......1<br>NO........0

DRAFT

**C**	Visit family or relatives					YES......1<br>NO........0→<br>***G6.05***		YES......1<br>NO........0→<br>PLACE D	IF I HAVE COMPANY (RELATIVES, CHILDREN)................................1<br>IF I CAN ARRANGE MY OWN EXPENSES (FOR TRANSPORT)............2<br>IF I FOLLOW PURDAH / DRESS ACCEPTABLY................................3<br>OTHER<br>(SPECIFY)...4<br>UNDER NO CIRCUMSTANCES WOULD I BE ALLOWED TO GO...........5 → ***PLACE D***	YES......1<br>NO........0
**D**	Visit a friend / neighbor's house					YES......1<br>NO........0→<br>***G6.05***		YES......1<br>NO........0→<br>PLACE E	IF I HAVE COMPANY (RELATIVES, CHILDREN)................................1<br>IF I CAN ARRANGE MY OWN EXPENSES (FOR TRANSPORT)............2<br>IF I FOLLOW PURDAH / DRESS ACCEPTABLY................................3<br>OTHER<br>(SPECIFY)...4<br>UNDER NO CIRCUMSTANCES WOULD I BE ALLOWED TO GO...........5 → ***PLACE E***	YES......1<br>NO........0
**E**	Hospital / clinic / doctor (seek health service)					YES......1<br>NO........0→<br>***G6.05***		YES......1<br>NO........0→<br>PLACE F	IF I HAVE COMPANY (RELATIVES, CHILDREN)................................1<br>IF I CAN ARRANGE MY OWN EXPENSES (FOR TRANSPORT)............2<br>IF I FOLLOW PURDAH / DRESS ACCEPTABLY................................3<br>OTHER<br>(SPECIFY)...4<br>UNDER NO CIRCUMSTANCES WOULD I BE ALLOWED TO GO...........5 → ***PLACE F***	YES......1<br>NO........0
**F**	Extension office/veterinarian					YES......1<br>NO........0→<br>***G6.05***		YES......1<br>NO........0→<br>PLACE G	IF I HAVE COMPANY (RELATIVES, CHILDREN)................................1<br>IF I CAN ARRANGE MY OWN EXPENSES (FOR TRANSPORT)............2<br>IF I FOLLOW PURDAH / DRESS ACCEPTABLY................................3<br>OTHER<br>(SPECIFY)...4<br>UNDER NO CIRCUMSTANCES WOULD I BE ALLOWED TO GO...........5 → ***PLACE G***	YES......1<br>NO........0
**G**	Business group meetings (SACCO meetings, etc.)					YES......1<br>NO........0→<br>***G6.05***		YES......1<br>NO........0→<br>PLACE H	IF I HAVE COMPANY (RELATIVES, CHILDREN)................................1<br>IF I CAN ARRANGE MY OWN EXPENSES (FOR TRANSPORT)............2<br>IF I FOLLOW PURDAH / DRESS ACCEPTABLY................................3<br>OTHER<br>(SPECIFY)...4<br>UNDER NO CIRCUMSTANCES WOULD I BE ALLOWED TO GO...........5 → ***PLACE H***	YES......1<br>NO........0

DRAFT

**H**	Training or capacity building in dairy production, handling, or sale					YES……1<br>NO……..0→<br>***G6.05***		YES……1<br>NO……..0→<br>PLACE I	IF I HAVE COMPANY (RELATIVES, CHILDREN)……………………………1<br>IF I CAN ARRANGE MY OWN EXPENSES (FOR TRANSPORT)…………..2<br>IF I FOLLOW PURDAH / DRESS ACCEPTABLY……………………………3<br>OTHER (SPECIFY)…………………………………………………………………4<br>UNDER NO CIRCUMSTANCES WOULD I BE ALLOWED TO GO………..5 → ***PLACE I***	YES……1<br>NO……..0
**I**	Somewhere other than your home for one or more nights					YES……1<br>NO……..0→<br>***G6.05***		YES……1<br>NO……..0→<br>PLACE J.	IF I HAVE COMPANY (RELATIVES, CHILDREN)……………………………1<br>IF I CAN ARRANGE MY OWN EXPENSES (FOR TRANSPORT)…………..2<br>IF I FOLLOW PURDAH / DRESS ACCEPTABLY……………………………3<br>OTHER (SPECIFY)…………………………………………………………………4<br>UNDER NO CIRCUMSTANCES WOULD I BE ALLOWED TO GO………..5 → ***PLACE J***	YES……1<br>NO……..0
**J**	Temple / church / mosque					YES……1<br>NO……..0→<br>***G6.05***		YES……1<br>NO……..0→<br>PLACE K.	IF I HAVE COMPANY (RELATIVES, CHILDREN)……………………………1<br>IF I CAN ARRANGE MY OWN EXPENSES (FOR TRANSPORT)…………..2<br>IF I FOLLOW PURDAH / DRESS ACCEPTABLY……………………………3<br>OTHER (SPECIFY)…………………………………………………………………4<br>UNDER NO CIRCUMSTANCES WOULD I BE ALLOWED TO GO………..5 → ***PLACE K***	YES……1<br>NO……..0
**K**	Public village gathering or community meeting					YES……1<br>NO……..0→<br>***G6.05***		YES……1<br>NO……..0→<br>G7.01.	IF I HAVE COMPANY (RELATIVES, CHILDREN)……………………………1<br>IF I CAN ARRANGE MY OWN EXPENSES (FOR TRANSPORT)…………..2<br>IF I FOLLOW PURDAH / DRESS ACCEPTABLY……………………………3<br>OTHER (SPECIFY)…………………………………………………………………4<br>UNDER NO CIRCUMSTANCES WOULD I BE ALLOWED TO GO………..5 → ***MODULE G7***	YES……1<br>NO……..0

**CODE G6.01**
EVERYDAY………………………………………………………………………………1
EVERY WEEK AT LEAST ONCE……………………………………………………2
EVERY 2 WEEKS AT LEAST ONCE…………………………………………………3
EVERY MONTH AT LEAST ONCE…………………………………………………4
LESS THAN ONCE A MONTH……………………………………………………5

**G6.04 RESPONSE CODES**	
1	TRANSPORTATION TOO EXPENSIVE
2	I DID NOT HAVE THE PROPER DRESS/CREDENTIALS
3	NOT ENOUGH TIME
4	I THOUGHT IT WAS UNSAFE
5	OTHERS TOLD ME IT WAS UNSAFE

DRAFT

NEVER......6	
NOT APPLICABLE 98	

6	FORBIDDEN TO GO BY SPOUSE/PARTNER
7	FORBIDDEN TO GO BY THE FAMILY OF MY SPOUSE/PATRNER
8	FORBIDDEN TO GO BY OWN FAMILY MEMBER
9	FORBIDDEN TO GO BY AN AUTHOIRTY
10	(DO NOT READ ALOUD) SOCIETAL NORM
11	OTHER, SPECIFY: _______________

**G6.08** How often do you go to a public village gathering / community meeting / training for NGO or programs?	
**G6.09.** In the last 12 months, how many times have you been away from home for one or more nights (in other words, sleeping somewhere else for the night)?	***IF RESPONDENT IS MALE →MODULE G7***

DRAFT

HOUSEHOLD ID						
RESPONDENT ID						

MODULE G7: INTRAHOUSEHOLD RELATIONSHIPS

DRAFT

Now I'd like to ask you some questions about how you feel about some of other people in your household or family group and how you think they feel about you.<br><br>**ENTER MEMBER ID FOR EACH RELATION**<br><br>**OTHER CODES:**<br>NON-HH MEMBER.......94			Do you [NAME] respect your [RELATION]?	Does your [RELATION] respect you?	Do you trust your [RELATION] to do things that are in your best interest?	When you disagree with your [RELATION], do you feel comfortable telling him/her that you disagree?	**IS [RELATION] THE OTHER RESPONDENT WITHIN THIS HOUSEHOLD?**	Is there a co-wife within your household?
**RELATION**			**G7.02**	**G7.03**	**G7.04**	**G7.05**	**G7.06**	**G7.07**
**A**	Husband / wife	**ID #**	MOST OF THE TIME......1<br>SOMETIMES ....2<br>RARELY..........................3<br>NEVER............................4	MOST OF THE TIME......1<br>SOMETIMES ....2<br>RARELY..........................3<br>NEVER............................4	MOST OF THE TIME......1<br>SOMETIMES ....2<br>RARELY..........................3<br>NEVER............................4	MOST OF THE TIME......1<br>SOMETIMES ....2<br>RARELY..........................3<br>NEVER............................4	YES......1 → ***RELATION C***<br>NO........0	
**B**	Other respondent within the household	**ID #**	MOST OF THE TIME......1<br>SOMETIMES ....2<br>RARELY..........................3<br>NEVER............................4	MOST OF THE TIME......1<br>SOMETIMES ....2<br>RARELY..........................3<br>NEVER............................4	MOST OF THE TIME......1<br>SOMETIMES ....2<br>RARELY..........................3<br>NEVER............................4	MOST OF THE TIME......1<br>SOMETIMES ....2<br>RARELY..........................3<br>NEVER............................4		
**C**	**IF RESPONDENT IS MALE:** Father (or adapt this category to capture other important relationship)<br><br>**IF RESPONDENT IS FEMALE:** Mother-in-law	**ID #**	MOST OF THE TIME......1<br>SOMETIMES ....2<br>RARELY..........................3<br>NEVER............................4	MOST OF THE TIME......1<br>SOMETIMES ....2<br>RARELY..........................3<br>NEVER............................4	MOST OF THE TIME......1<br>SOMETIMES ....2<br>RARELY..........................3<br>NEVER............................4	MOST OF THE TIME......1<br>SOMETIMES ....2<br>RARELY..........................3<br>NEVER............................4<br>***IF RESPONDENT IS MALE → MODULE G8(A)***		YES......1<br>NO........0 →<br>***MODULE G8(A)***
**D**	Most senior co-wife (the person who was in the household just before you, or, if you are the senior wife, the one who married into the household after you)	**ID #**	MOST OF THE TIME......1<br>SOMETIMES ....2<br>RARELY..........................3<br>NEVER............................4	MOST OF THE TIME......1<br>SOMETIMES ....2<br>RARELY..........................3<br>NEVER............................4	MOST OF THE TIME......1<br>SOMETIMES ....2<br>RARELY..........................3<br>NEVER............................4	MOST OF THE TIME......1<br>SOMETIMES ....2<br>RARELY..........................3<br>NEVER............................4		

HOUSEHOLD ID						
RESPONDENT ID						

MODULE G8(A): AUTONOMY IN DECISION-MAKING

DRAFT

Now I am going to read you some stories about different farmers and their situations regarding different agricultural activities. This question format is different from the rest so take your time in answering. For each I will then ask you how much you are like or not like each of these people. We would like to know if you are completely different from them, similar to them, or somewhere in between. There are no right or wrong answers to these questions.<br><br>**READ ALOUD EACH STORY, SUBSEQUENT QUESTIONs, AND RESPONSE CODES. NAMES SHOULD BE ADOPTED TO LOCAL CONTEXT AND BE MALE/FEMALE DEPENDING ON THE SEX OF THE RESPONDENT. THE ORDER OF TOPICS A-D SHOULD BE RANDOMIZED, AND WITHIN EACH TOPIC, THE ORDER OF STORIES 1-4 SHOULD BE RANDOMIZED.**			Are you like this person?<br><br>**CIRCLE ONE**	Are you completely the same or somewhat the same?<br><br>**CIRCLE ONE**	Are you completely different or somewhat different?<br><br>**CIRCLE ONE**
**STORY**			**G8.01**	**G8.02**	**G8.03**
Livestock raising	**B1**	*"[PERSON'S NAME] cannot raise any livestock other than what she has. These are all that do well here."*	YES...1<br>NO.....0 →<br>***G8.03***	COMPLETELY THE SAME....1 → ***B2***<br>SOMEWHAT THE SAME.......2 → ***B2***	COMPLETELY DIFFERENT....1<br>SOMEWHAT DIFFERENT.......2
	**B2**	*"[PERSON'S NAME] raises the types of livestock she does because her spouse, or another person or group in her community tell her she must use these breeds. She does what they tell her to do."*	YES...1<br>NO.....0 →<br>***G8.03***	COMPLETELY THE SAME....1 → ***B3***<br>SOMEWHAT THE SAME.......2 → ***B3***	COMPLETELY DIFFERENT....1<br>SOMEWHAT DIFFERENT.......2
	**B3**	*"[PERSON'S NAME] raises the kinds of livestock that her family or community expect. She wants them to approve of her as a good livestock raiser."*	YES...1<br>NO.....0 →<br>***G8.03***	COMPLETELY THE SAME....1 → ***B4***<br>SOMEWHAT THE SAME.......2 → ***B4***	COMPLETELY DIFFERENT....1<br>SOMEWHAT DIFFERENT.......2
	**B4**	*"[PERSON'S NAME] chooses the types of livestock that she personally wants to raise and thinks are good for herself and her family. She values raising these types. If she changed her mind, she could act differently."*	YES...1<br>NO.....0 →<br>***G8.03***	COMPLETELY THE SAME....1 → ***C1***<br>SOMEWHAT THE SAME.......2 → ***C1***	COMPLETELY DIFFERENT....1<br>SOMEWHAT DIFFERENT.......2

**READ ALOUD EACH STORY, SUBSEQUENT QUESTIONs, AND RESPONSE CODES. NAMES SHOULD BE ADOPTED TO LOCAL CONTEXT AND BE MALE/FEMALE DEPENDING ON THE SEX OF THE RESPONDENT.**			Are you like this person?<br><br>**CIRCLE ONE**	Are you completely the same or somewhat the same?<br><br>**CIRCLE ONE**	Are you completely different or somewhat different?<br><br>**CIRCLE ONE**
**STORY**			**G8.01**	**G8.02**	**G8.03**
Taking crops or livestock	**C1**	*"There is no alternative to how much or how little of her crops or livestock [PERSON'S NAME] can take to the market. She is taking the only possible amount."*	YES...1<br>NO.....0 →<br>***G8.03***	COMPLETELY THE SAME....1 → ***C2***<br>SOMEWHAT THE SAME.......2 → ***C2***	COMPLETELY DIFFERENT....1<br>SOMEWHAT DIFFERENT.......2

(incl. eggs or milk) to the market (or not)	C2	*"[PERSON'S NAME] takes crops and livestock to the market because her spouse, or another person or group in her community tell her she must sell them there. She does what they tell her to do."*	YES...1<br>NO.....0 → ***G8.03***	COMPLETELY THE SAME....1 → ***C3***<br>SOMEWHAT THE SAME.......2 → ***C3***	COMPLETELY DIFFERENT....1<br>SOMEWHAT DIFFERENT.......2
	C3	*"[PERSON'S NAME] takes the crops and livestock to the market that her family or community expect. She wants them to approve of her."*	YES...1<br>NO.....0 → ***G8.03***	COMPLETELY THE SAME....1 → ***C4***<br>SOMEWHAT THE SAME.......2 → ***C4***	COMPLETELY DIFFERENT....1<br>SOMEWHAT DIFFERENT.......2
	C4	*"[PERSON'S NAME] chooses to take the crops and livestock to market that she personally wants to sell there, and thinks is best for herself and her family. She values this approach to sales. If she changed her mind, she could act differently."*	YES...1<br>NO.....0 → ***G8.03***	COMPLETELY THE SAME....1 → ***D1***<br>SOMEWHAT THE SAME.......2 → ***D1***	COMPLETELY DIFFERENT....1<br>SOMEWHAT DIFFERENT.......2
How to use income generated from agricultural and non-agricultural activities	D1	*"There is no alternative to how [PERSON'S NAME] uses her income. How she uses her income is determined by necessity."*	YES...1<br>NO.....0 → ***G8.03***	COMPLETELY THE SAME....1 → ***D2***<br>SOMEWHAT THE SAME.......2 → ***D2***	COMPLETELY DIFFERENT....1<br>SOMEWHAT DIFFERENT.......2
	D2	*"[PERSON'S NAME] uses her income how her spouse, or another person or group in her community tell her she must use it there. She does what they tell her to do."*	YES...1<br>NO.....0 → ***G8.03***	COMPLETELY THE SAME....1 → ***D3***<br>SOMEWHAT THE SAME.......2 → ***D3***	COMPLETELY DIFFERENT....1<br>SOMEWHAT DIFFERENT.......2
	D3	*"[PERSON'S NAME] uses her income in the way that her family or community expect. She wants them to approve of her."*	YES...1<br>NO.....0 → ***G8.03***	COMPLETELY THE SAME....1 → ***D4***<br>SOMEWHAT THE SAME.......2 → ***D4***	COMPLETELY DIFFERENT....1<br>SOMEWHAT DIFFERENT.......2
	D4	*"[PERSON'S NAME] chooses to use her income how she personally wants to, and thinks is best for herself and her family. She values using her income in this way. If she changed her mind, she could act differently."*	YES...1<br>NO.....0 → ***G8.03***	COMPLETELY THE SAME...1→***G8.04***<br>SOMEWHAT THE SAME....2 →***G8.04***	COMPLETELY DIFFERENT....1<br>SOMEWHAT DIFFERENT.......2

DRAFT

MODULE G8(B): NEW GENERAL SELF-EFFICACY SCALE

Now I'm going to ask you some questions about different feelings you might have. Please listen to each of the following statements. Think about how each statement relates to your life, and then tell me how much you agree or disagree with the statement on a scale of 1 to 5, where 1 means you "strongly disagree" and 5 means you "strongly agree." (**Note: Randomize order of statements**)

STATEMENTS		G8.04
**A**	I will be able to achieve most of the goals that I have set for myself.	STRONGLY DISAGREE .... 1<br>DISAGREE .... 2<br>NEITHER AGREE NOR DISAGREE .... 3<br>AGREE .... 4<br>STRONGLY AGREE 5
**B**	When facing difficult tasks, I am certain that I will accomplish them.	STRONGLY DISAGREE .... 1<br>DISAGREE .... 2<br>NEITHER AGREE NOR DISAGREE .... 3<br>AGREE .... 4<br>STRONGLY AGREE 5
**C**	In general, I think that I can obtain outcomes that are important to me.	STRONGLY DISAGREE .... 1<br>DISAGREE .... 2<br>NEITHER AGREE NOR DISAGREE .... 3<br>AGREE .... 4<br>STRONGLY AGREE 5
**D**	I believe I can succeed at most any endeavor to which I set my mind	STRONGLY DISAGREE .... 1<br>DISAGREE .... 2<br>NEITHER AGREE NOR DISAGREE .... 3<br>AGREE .... 4<br>STRONGLY AGREE 5
**E**	I will be able to successfully overcome many challenges.	STRONGLY DISAGREE .... 1<br>DISAGREE .... 2<br>NEITHER AGREE NOR DISAGREE .... 3<br>AGREE .... 4<br>STRONGLY AGREE 5
**F**	I am confident that I can perform effectively on many different tasks.	STRONGLY DISAGREE .... 1<br>DISAGREE .... 2<br>NEITHER AGREE NOR DISAGREE .... 3<br>AGREE .... 4<br>STRONGLY AGREE 5
**G**	Compared to other people, I can do most tasks very well.	STRONGLY DISAGREE .... 1<br>DISAGREE .... 2<br>NEITHER AGREE NOR DISAGREE .... 3<br>AGREE .... 4<br>STRONGLY AGREE 5
**H**	Even when things are tough, I can perform quite well.	STRONGLY DISAGREE .... 1<br>DISAGREE .... 2<br>NEITHER AGREE NOR DISAGREE 3

DRAFT

		AGREE .... 4<br>STRONGLY AGREE 5

MODULE G8(C): LIFE SATISFACTION

The following questions ask how satisfied you feel with your life as a whole, on a scale from 1 to 5, where 1 means you feel "very dissatisfied" and 5 means you feel "very satisfied."		
	**STATEMENTS**	**G8.05**
**A**	Overall, how satisfied are you with life as a whole these days?	VERY DISSATISFIED....1<br>DISSATISFIED....2<br>NEITHER SATISFIED NOR DISSATISFIED....3<br>SATISFIED....4<br>VERY SATISFIED....5
**B**	Overall, how satisfied with your life were you 5 years ago?	VERY DISSATISFIED....1<br>DISSATISFIED....2<br>NEITHER SATISFIED NOR DISSATISFIED....3<br>SATISFIED....4<br>VERY SATISFIED....5
**C**	As your best guess, overall how satisfied with your life do you expect to feel 5 years from today?	VERY DISSATISFIED....1<br>DISSATISFIED....2<br>NEITHER SATISFIED NOR DISSATISFIED....3<br>SATISFIED....4<br>VERY SATISFIED....5

DRAFT

HOUSEHOLD ID						
RESPONDENT ID						

MODULE G9. Attitudes about Domestic Violence

	Now I would like to ask about your opinion on the following issues. Please keep in mind that I am not asking about your personal experience or whether the following scenarios have happened to you. I would only like to know whether you think the following issues are acceptable.	In your opinion, is a husband justified in hitting or beating his wife in the following situations?
**SITUATION**		**G9.01**
**A**	If she goes out without telling him?	YES....1<br>NO....0<br>DON'T KNOW....88
**B**	If she neglects the children?	YES....1<br>NO....0<br>DON'T KNOW....88
**C**	If she argues with him?	YES....1<br>NO....0<br>DON'T KNOW....88
**D**	If she refuses to have sex with him?	YES....1<br>NO....0<br>DON'T KNOW....88
**E**	If she burns the food?	YES....1<br>NO....0<br>DON'T KNOW....88
**F**	If some livestock is lost	YES....1<br>NO....0<br>DON'T KNOW....88
**G**	If she sells one livestock without consulting	YES....1<br>NO....0<br>DON'T KNOW....88

END OF QUESTIONAIRE

DRAFT
